# Ensemble-imbalance-based classification for amyotrophic lateral sclerosis prognostic prediction: identifying short-survival patients at diagnosis

**DOI:** 10.1186/s12911-024-02484-5

**Published:** 2024-03-19

**Authors:** Fabiano Papaiz, Mario Emílio Teixeira Dourado, Ricardo Alexsandro de Medeiros Valentim, Rafael Pinto, Antônio Higor Freire de Morais, Joel Perdiz Arrais

**Affiliations:** 1 Federal University of Rio Grande Do Norte, Natal, Brazil; 2 University of Coimbra, Coimbra, Portugal; 3 Federal Institute of Rio Grande Do Norte, Natal, Brazil

**Keywords:** Amyotrophic lateral sclerosis, Prognosis, Machine learning, Health informatics

## Abstract

Prognosticating Amyotrophic Lateral Sclerosis (ALS) presents a formidable challenge due to patients exhibiting different onset sites, progression rates, and survival times. In this study, we have developed and evaluated Machine Learning (ML) algorithms that integrate Ensemble and Imbalance Learning techniques to classify patients into Short and Non-Short survival groups based on data collected during diagnosis. We aimed to identify individuals at high risk of mortality within 24 months of symptom onset through analysis of patient data commonly encountered in daily clinical practice. Our Ensemble-Imbalance approach underwent evaluation employing six ML algorithms as base classifiers. Remarkably, our results outperformed those of individual algorithms, achieving a Balanced Accuracy of 88% and a Sensitivity of 96%. Additionally, we used the Shapley Additive Explanations framework to elucidate the decision-making process of the top-performing model, pinpointing the most important features and their correlations with the target prediction. Furthermore, we presented helpful tools to visualize and compare patient similarities, offering valuable insights. Confirming the obtained results, our approach could aid physicians in devising personalized treatment plans at the time of diagnosis or serve as an inclusion/exclusion criterion in clinical trials.

**Supplementary Information:**

The online version contains supplementary material available at 10.1186/s12911-024-02484-5.

## Introduction

Amyotrophic Lateral Sclerosis (ALS) is a rare, incurable, and progressive neurodegenerative disease that impacts the human motor system. The communication between the brain and muscles gradually deteriorates, ultimately resulting in paralysis and death. While its etiology remains unknown, it typically afflicts individuals aged between 40 and 70, affecting both men and women. ALS exhibits significant clinical heterogeneity, manifesting diverse symptoms and disease progression patterns among patients [1, 2]. The average life expectancy post-symptom onset ranges from 2 to 5 years, with a global annual incidence of approximately 1.9 cases per 100,000 individuals [3]. Given the complexity of ALS and its variable clinical presentation, accurately predicting outcomes such as survival time and disease progression rate poses a substantial challenge for physicians. Therefore, it is imperative to conduct research aiming to develop and validate prognostic models to achieve more precise predictive results.

Machine Learning (ML) has emerged as a powerful tool in improving disease diagnosis and prognosis. In the context of ALS, recent studies have explored various ML approaches for diverse predictive tasks [4–7]. ML algorithms can extract information from training data, convert it to knowledge, and apply it to solve various types of problems, such as classification, regression, and clustering [8]. Learning from complex domains (e.g., ALS disease) is challenging, and ML techniques like Ensemble Learning can help improve predictive performance. Ensemble Learning combines single predictive models to build a more complex one, aiming to surpass the performance of each constituent model separately [9]. Additionally, medical data analysis often involves dealing with imbalanced datasets. This issue arises when there is a significant imbalance in the number of samples between different classes, resulting in a substantially lower representation of one class compared to the others. Typically, the target prediction is linked to samples from the minority class, as in the case of detecting patients with lung cancer through the analysis of tomography images, where there are significantly more images of healthy patients (the majority class) than those depicting lung cancer cases (the minority class). In such scenarios, ML models frequently exhibit bias towards samples belonging to the majority class, resulting in an elevated misclassification rate within the minority class [10]. To mitigate the issue of imbalance, resampling techniques such as Undersampling and Oversampling can be employed [11].

Efforts in ALS prognosis using ML should be directed toward the development of Clinical Decision Support (CDS) systems. CDS systems are computer programs designed to assist healthcare professionals in making more informed and timely decisions by integrating current patient data with historical information from other patients, facilitating data-driven decision making [12]. We deemed it essential to develop CDS systems that are feasible for use on a large scale in primary care, taking into account financial limitations. One approach to achieving this goal is to select biological markers (biomarkers) commonly used in routine ALS clinical practice. Such biomarkers may include clinical evaluations, assessment of functional capabilities, and respiratory function measurements. These biomarkers are often derived from less expensive and complex procedures, making them more accessible.

Notably, some ML algorithms present results that humans cannot easily understand, decreasing their interpretability (e.g., Artificial Neural Networks or Support Vector Machines). Interpretability, in this context, refers to the comprehensibility of the decisions made by the ML algorithm [13]. Addressing this issue is crucial to ensure the acceptance of CDS systems in clinical practice, as physicians require explanations for patient classifications. Hence, the development of CDS systems must prioritize interpretability concerns. Existing frameworks can be explored to elucidate the predictions generated by ML models, one of which is the Shapley Additive Explanations (SHAP) framework [14]. SHAP employs a game-theoretic approach to clarify the prediction for a specific instance by quantifying the contribution (SHAP value) of each feature to the classification process. Consequently, SHAP values provide insights into the influence of individual features on the final prediction and their relative significance when compared to other features.

### Related work

Van der Burgh et al. [4] demonstrated the positive impact of using Magnetic Resonance Images (MRI) and clinical information to classify ALS patients into survival groups (Short, Medium, and Long). They developed Deep Neural Networks models and obtained an accuracy of 84%. This study presented a high risk of model overfitting due to the reduced number of samples analyzed (*n* = 135). Kueffner et al. [15] presented a crowdsourcing challenge involving more than 30 teams. One of the target predictions was the probability of survival at 12-, 18-, and 24-months using patient data from the first three months of records. A team using a Gaussian Process Regression model obtained the best performance compared to the others (Z-score ≈ 12). These studies used different performance metrics, and thus, it was not possible to directly compare their performance with this study.

Grollemund et al. [16] presented a model based on Dimensionality Reduction to predict one-year survival probabilities. They used the Uniform Manifold Approximation and Projection (UMAP) algorithm to reduce the data. The resultant 2D projection was divided into three areas to classify the patients into Low, Intermediate, and High probabilities groups. The proposed classifier obtained superior performance (F1 score: 96%, Balanced Accuracy: 91%) than the Random Forest and Logistic Regression models. However, the total comprehension of the relationship between input and output variables cannot be obtained because the adopted model is considered a black box approach, which degrades its interpretability. In this study, we achieved a slightly lower Balanced Accuracy (88%) and evaluated models that were also considered black boxes. Differently, we delivered global and local explanations regarding the prediction mechanisms, including feature importance analysis and their correlations with the target variable.

Tavazzi et al. [17] presented a strategy based on a mutual information-weighted k-NN algorithm to handle missing values in clinical register datasets. One target was to classify patients into Short and Long survival groups. The authors evaluated Naïve Bayes classifiers built on the dataset using the proposed imputation method and achieved a superior performance (AUC: 82%) compared to classifiers using other imputation approaches. We achieved a superior AUC (93%) in this study, which suggests a greater effectiveness of our approach.

### Our contribution

In this study, we have delved into the utilization of Ensemble and Imbalance Learning techniques to enhance the prediction accuracy for ALS patients with short survival expectancy. Our primary aim was to classify patients into Short and Non-Short survival groups based on data collected at the time of diagnosis. The Short survival group comprises individuals who die within 24 months from the onset of symptoms, indicating a rapid disease progression rate. This 24-month threshold was chosen based on the typical life expectancy of ALS patients, which ranges from 2 to 5 years. Hence, our goal was to identify patients in critical condition during diagnosis. This classification is essential for providing timely information to patients and their families, improving the quality of end-of-life care, and facilitating treatment and resource planning. The analyzed dataset showed a significant data imbalance, with 13% representing the minority class (Short) and 87% representing the majority class (Non-Short). Our focus was centered on the examination of biomarkers commonly encountered in routine ALS clinical practice.

The proposed solution combined Ensemble and Imbalance learning techniques to improve the prediction of critical ALS patients at diagnosis time. Our Ensemble-Imbalance approach obtained the best performance, achieving a Balanced Accuracy of 88% and a Sensitivity of 96% using a Neural Network model as the base classifier. Furthermore, we employed the SHAP framework to provide insights into how the best model conducted patient classifications.

The principal contributions of our study encompass: (i) the development and evaluation of models through an Ensemble-Imbalance-based approach, resulting in improved performance in identifying critically affected ALS patients at the time of diagnosis, (ii) delivering both global and local explanations regarding the model’s prediction mechanisms, including the identification of pivotal features and their correlations with the target variable, and (iii) offering an effective preprocessing methodology for ALS patient data that enabled the extraction of relevant ALS characteristics using biomarkers commonly encountered in clinical practice.

## Methods

To ensure the systematic execution of our experiments, we organized our models into two distinct scenarios: Single-Model and Ensemble-Imbalance. In the initial phase, we designed and evaluated models using state-of-the-art machine learning algorithms, including k-Nearest Neighbors (k-NN), Decision Tree (DT), Random Forest (RF), Support Vector Machines (SVM), Naïve Bayes (NB), and Neural Networks (NN). These models constituted the Single-Model scenario. Subsequently, we utilized the top ten models for each algorithm as base classifiers to develop and evaluate the Ensemble-Imbalance-based models. Following this, we selected the best-performing model for each algorithm and scenario and conducted a comparative analysis of their results (see Supplementary Information for more details). Finally, we employed the SHAP framework to elucidate how the overall best model executed patient classifications, offering insights into the significance of each feature, in addition to providing both global and local interpretability of the model.

The data analyzed in this study can be accessed from the PRO-ACT website (https://ncri1.partners.org/ProACT). It is important to note that data derived from this database cannot be shared due to restrictions. However, comprehensive details about the source code employed in this study, including data preprocessing, model development, hyperparameter settings, and software versions, are available at the public repository https://github.com/fabianopapaiz/ensemble_imbalance_model_for_als_prognosis.

### Patient data

All data used in this article were sourced from the Pooled Resource Open-Access ALS Clinical Trials Database (PRO-ACT) [18]. PRO-ACT is the largest open-access dataset available for ALS disease. It comprises over 11,600 records, which contain historical data from 29 clinical trials on ALS. The dataset provides information on clinical, functional, respiratory, laboratory exams, death reports, and other biomarkers. The data available in the PRO-ACT Database have been volunteered by PRO-ACT Consortium members.

For the purposes of our study, we extracted a range of pertinent information, including demographic details (age, weight, height, and gender), the administration of the Riluzole drug, familial medical history, Forced Vital Capacity (FVC; expressed as a percentage of normal for a healthy individual, adjusted for gender, age, and height), Slow Vital Capacity (SVC), Body Mass Index (BMI), El Escorial diagnostic criteria, ALS Functional Rate Scale (ALSFRS), and Revised ALS Functional Rate Scale (ALSFRS-R). The ALSFRS scale comprises ten inquiries focused on assessing various physical functionalities, such as speech, swallowing, handwriting, turning in bed, walking, climbing stairs, and respiratory [19]. The ALSFRS-R scale replaced the single respiratory function question with three more detailed questions [20].

### Design of the experiments

**Fig. 1:**
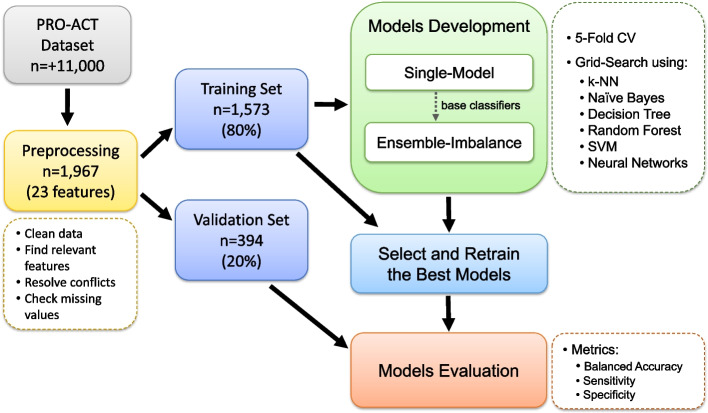
ML pipeline design to execute the experiments of this study

We put forward an ML pipeline divided into the stages as shown in Fig. 1, which were detailed hereafter. All experiments were performed using the Python programming language and packages for data analysis, machine learning, and data visualization, including Pandas, Scikit-Learn, Imbalance-Learning, Matplotlib, Seaborn, SHAP, and NumPy.

#### Data preprocessing

Patient data collected during diagnosis was analyzed, as previously mentioned. To facilitate their analysis, temporal features were transformed into static data through a technique known as Summary Measures. This approach offers several advantages, including simplicity of interpretation, compatibility with uneven time intervals between measurements, and statistical robustness and validity [21]. We utilized values recorded on the date of diagnosis for temporal features such as FVC, SVC, and BMI. In instances where these values were unavailable, we selected the measurement closest to the diagnosis date for the respective samples.

As recommended in our previous study [22], we analyzed the slope of each ALSFRS question separately instead of the total slope. This approach enabled us to perform a more granular examination of functional loss characteristics among patients, aiding in the identification of the most pertinent ALSFRS questions for our target prediction. Gordon and Lerner [6] presented an approach to merge data from both ALSFRS and ALSFRS-R scales by combining the samples using only information about *Dyspnea* (question 10) for those assessed with the ALSFRS-R scale. This enabled them to convert the ALSFRS-R scale to ALSFRS, thereby expanding the sample size. In alignment with this approach, we adopted the same strategy in this study, as 51% of the PRO-ACT samples were assessed using the ALSFRS-R scale. Consequently, questions 11 and 12 of the ALSFRS-R scale were not included in our analysis. To model the ALSFRS questions as non-temporal variables, we summarized their data into single slope values. These slopes were calculated as depicted in Eq. (1), where *4* represents the maximum question score, *Question Score at Diagnosis* denotes the score assessed at (or closest to) the time of diagnosis, and *Disease Duration* is the time in months between symptom onset and the time of diagnosis.

1 $${\text{Slope}}=\frac{4-Question\_Score\_at\_Diagnosis}{Disease\_Duration}$$

Additional features were created to store information about the age at symptom onset, the BMI, and whether the patient deceased within 24 months from symptom onset (Survival Group). The age at onset was calculated using information about the age at diagnosis and the disease duration. The BMI was calculated using the patient weight and height collected at the diagnosis. The Survival Group feature was used to classify patients with respect to the target prediction, i.e., into the Short and Non-Short survival groups. The Short survival group included patients who died within 24 months from the onset of the symptoms. We excluded patients whose last visit was within 24 months of disease onset and who were not marked as deceased in the PRO-ACT database.

We performed a complete case analysis, whereby our preprocessed dataset comprised solely of samples with no missing feature values. Features exhibiting a significant percentage of missing values were excluded: SVC (87%) and El Escorial (71%). This action was imperative to prevent the loss of a substantial number of samples. Before being used by the ML models, the features were scaled to a range between 0 and 1, and the dataset was partitioned into Training and Validation subsets. We allocated 80% of the samples for training the models and reserved 20% for validation.

#### Models development

This phase encompassed two key steps: (i) splitting the training data using a 5-fold Cross-Validation (CV) repeated three times and (ii) executing the models using a grid search strategy. We developed models using the following ML algorithms: k-NN, NB, DT, RF, SVM, and NN. In the Single-Model scenario, the models were directly executed using the 5-fold CV strategy in conjunction with diverse hyperparameter configurations as part of the grid search.

**Fig. 2:**
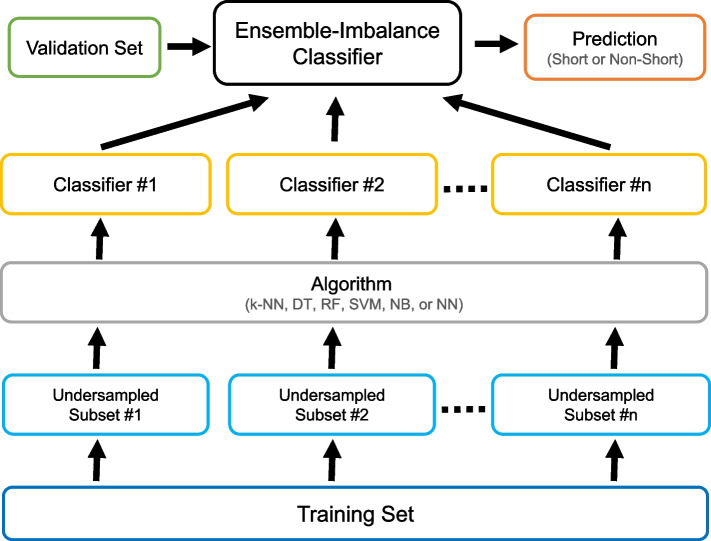
Overview of the Ensemble-Imbalance classifier proposed in this study. First, independent undersampled subsets are generated from the Training set using the Random Undersampling method. Then, classifiers created using a specific ML algorithm learn from these subsets (each classifier accesses only one subset). Finally, a majority voting strategy is used to classify patients into survival groups

In the Ensemble-Imbalance scenario, the models were executed using the following classifiers: Balanced Bagging (DT, SVM, NN, NB, and k-NN) and Balanced Random Forest (RF). These classifiers integrate Ensemble and Resampling techniques to increase the classification performance for minority classes without a significant decrease for the majority class. Initially, this approach creates multiple independent subsets of the original training data. Then, the number of samples in the different classes is equalized for each subset by randomly removing samples from the majority class using the Random Undersampling [11] method. Finally, instances of the same base classifier are trained using each of these subsets, and the final prediction is computed using a voting or averaging mechanism. Figure 2 provides an overview of the Ensemble-Imbalance classifier proposed in this study. The ten best classifiers for each algorithm obtained from the Single-Model scenario were utilized as base classifiers to create the models of the Ensemble-Imbalance scenario.

#### Selecting and retraining the best models

The top-performing models were chosen based on their Balanced Accuracy achieved using the Training set in both scenarios. Afterward, the best models were retrained (refitted) using the Training set and used to make predictions by accessing the Validation set. All obtained validation performance metrics were recorded and subjected to subsequent analysis and comparison.

#### Models evaluation

In our evaluation and comparative analysis of all ML models, we employed the following metrics in this order: Balanced Accuracy, Sensitivity, and Specificity. Sensitivity and Specificity were utilized as they signify the proportion of correctly classified Short and Non-Short survival patients, respectively. It is worth noting that Sensitivity held greater significance than Specificity in our evaluation, given our priority was to correctly classify Short survival patients, as they represent the critical cases. Balanced Accuracy was selected as the appropriate metric for evaluating the experiments as it represents the arithmetic average of Sensitivity and Specificity. Consequently, a higher Balanced Accuracy signifies superior predictive performance concerning both groups of patients.

We applied the Bonferroni correction method to ascertain whether the performance attained in the Ensemble-Imbalance scenario significantly surpassed that of the Single-Model scenario for each algorithm. It is essential to counteract the multiple comparisons problem due to the number of executions using 5-Fold CV repeated three times.

#### Feature importance and model explanation

Following the evaluation and identification of the best overall model (specifically, the Ensemble-Imbalance-based model utilizing NN as the base classifier), we conducted an in-depth analysis of how this model classifies patients using the SHAP framework. We detailed the significance of each feature for the classification process in the results section, providing comprehensive insights into global and local interpretability. To generate SHAP values and explanations, we employed the *Kernel-Explainer* class. All SHAP graphs were produced using the functionalities provided by this framework.

## Results

### Data preprocessing

This study accessed ALS patient data from the PRO-ACT database. Despite its large number of samples (over 11,600), we used only 17% of the available data. We opted to perform a complete case analysis, which reduced the number of samples that could be included due to a high percentage of missing values. The preprocessed dataset encompassed 1,967 patients, each characterized by 23 features. This dataset exhibited an Imbalance Ratio of 6.9 concerning the distribution of the minority and majority classes, with Short survival comprising 13% and Non-Short constituting 87% of the cases. Table 1 provides a comprehensive overview of all features analyzed in this study, along with their respective values and distributions.

**Table 1:** Features analyzed in this study with details on overall distribution and by survival group

Features		Values	All Samples	Short (13%)	Non-Short (87%)	Temporal
Diagnostic Delay (Disease Duration)		• Average (9 − 18 months) • Short (≤ 8 months) • Long (≥ 19 months)	40% 39% 21%	29% 66% 5%	41% 35% 24%	No
Age at Onset (range)		• 0 − 39 • 40 − 49 • 50 − 59 • 60 − 69 • 70 +	13% 22% 31% 26% 8%	4% 16% 35% 31% 14%	15% 22% 31% 25% 7%	
Sex		• Female • Male	36% 64%	30% 70%	37% 63%	
Site of Onset		• Bulbar • Limb/Spinal	19% 81%	26% 74%	18% 82%	
Riluzole		• No • Yes	69% 31%	74% 26%	68% 32%	
Forced Vital Capacity (FVC)		• Abnormal (< 80%) • Normal (≥ 80%)	21% 79%	32% 68%	20% 80%	Yes
Body Mass Index (BMI)		• Underweight (≤ 18*.*4) • Normal (18*.*5 − 24*.*9) • Overweight (25*.*0 − 29*.*9) • Obesity (≥ 30)	3% 37% 37% 23%	3% 40% 38% 19%	3% 37% 37% 23%	
Gastrostomy		• No • Yes	96% 4%	97% 3%	96% 4%	
Regions Involved	Quantity	• 1 • 2 • 3 • 4	13% 28% 34% 25%	10% 25% 36% 29%	14% 28% 33% 25%	
	Bulbar	• No • Yes	35% 65%	29% 71%	36% 64%	
	Upper Limb		19% 81%	20% 80%	19% 81%	
	Lower Limb		13% 87%	13% 87%	13% 87%	
	Respiratory		62% 38%	54% 46%	63% 37%	
ALSFRS Slopes by Question	Q1 − Speech	• Average (0*.*05 − 0*.*13*/month*) • Rapid (≥ 0*.*14*/month*) • Slow (≤ 0*.*04*/month*)	18% 3% 79%	34% 10% 56%	16% 2% 82%	
	Q2 − Salivation		12% 2% 86%	26% 7% 67%	10% 1% 89%	
	Q3 − Swallowing		13% 1% 86%	29% 4% 67%	10% 1% 89%	
	Q4 − Handwriting		18% 3% 79%	30% 12% 58%	16% 1% 83%	
	Q5 − Cutting		24% 4% 72%	31% 17% 52%	23% 2% 75%	
	Q6 − Dressing & Hygiene		30% 5% 65%	42% 21% 37%	28% 3% 69%	
	Q7 − Turning in Bed		18% 2% 80%	33% 11% 56%	16% 1% 83%	
	Q8 − Walking		29% 3% 68%	43% 16% 41%	27% 2% 71%	
	Q9 − Climbing Stairs		37% 11% 52%	36% 35% 29%	38% 8% 54%	
	Q10 − Respiratory		8% 1% 91%	20% 4% 76%	6% 1% 93%	

### Performance obtained by algorithm and scenario

**Fig. 3:**
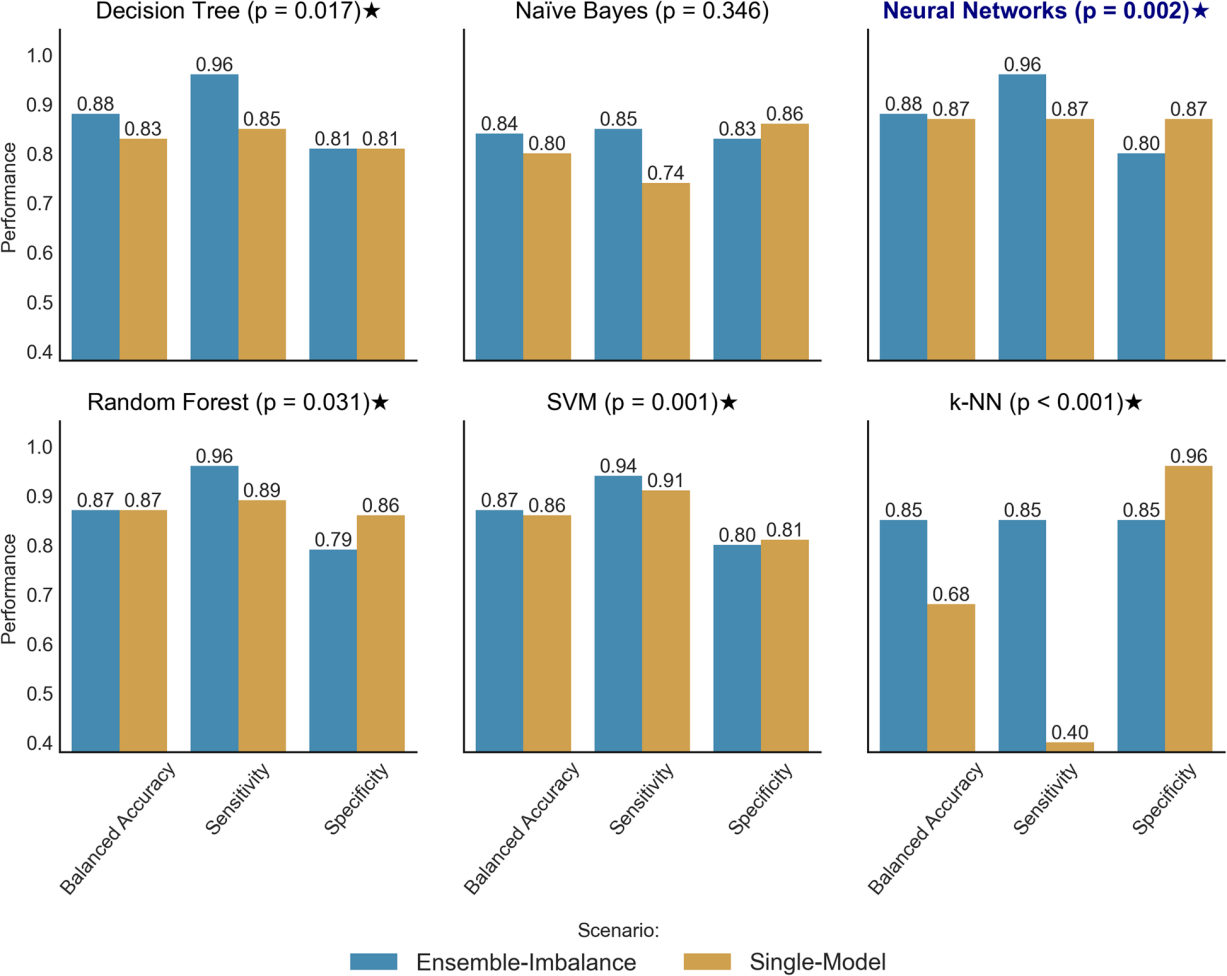
Comparison of the best performances obtained by each algorithm and scenario. The “p” represents the *p* value obtained after applying the Bonferroni correction method to compare the performance in both scenarios for each algorithm. Algorithms presenting a significantly better performance in the Ensemble Imbalance scenario were highlighted using the ⋆ symbol. Neural Networks performed significantly better (*p*-value ≤ 0.001) than the others in the Ensemble-Imbalance scenario (highlighted in bold and blue font)

Figure 3 visually depicts the top validation performances achieved by each algorithm and scenario. The “p” alongside each algorithm’s name indicates the *p*-value calculated after employing the Bonferroni correction method. Algorithms that exhibited significantly improved performance in the Ensemble-Imbalance scenario were denoted by the ⋆ symbol. We applied the same method to compare the performance of algorithms in the Ensemble-Imbalance scenario. The Neural Networks outperformed significantly (*p*-value ≤ 0.001) the others (DT, RF, SVM, and k-NN).

### Feature importance and model explanation

**Fig. 4:**
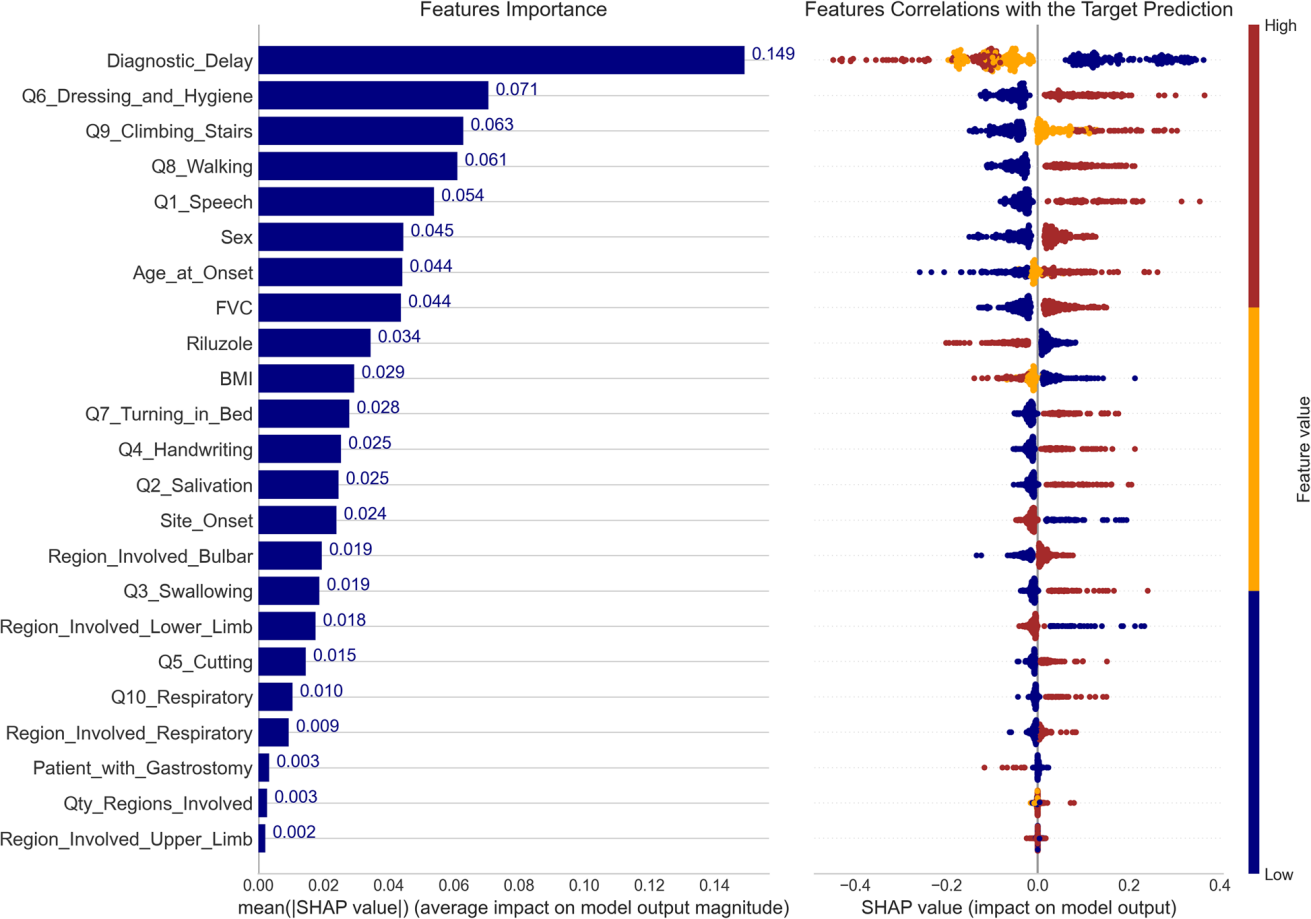
Ranking of feature importance and their correlations with the target variable (Short/Non-Short) for the best model in the Ensemble-Imbalance scenario. The x-axis on the left displays the average impact on the model prediction for each feature (mean absolute SHAP value). The x-axis on the right demonstrates the impact on the model prediction (SHAP value) concerning the feature values. Positive SHAP values indicate that the feature value led the model prediction toward the Short survival group, while negative SHAP values pushed it toward the Non-Short group

**Fig. 5:**
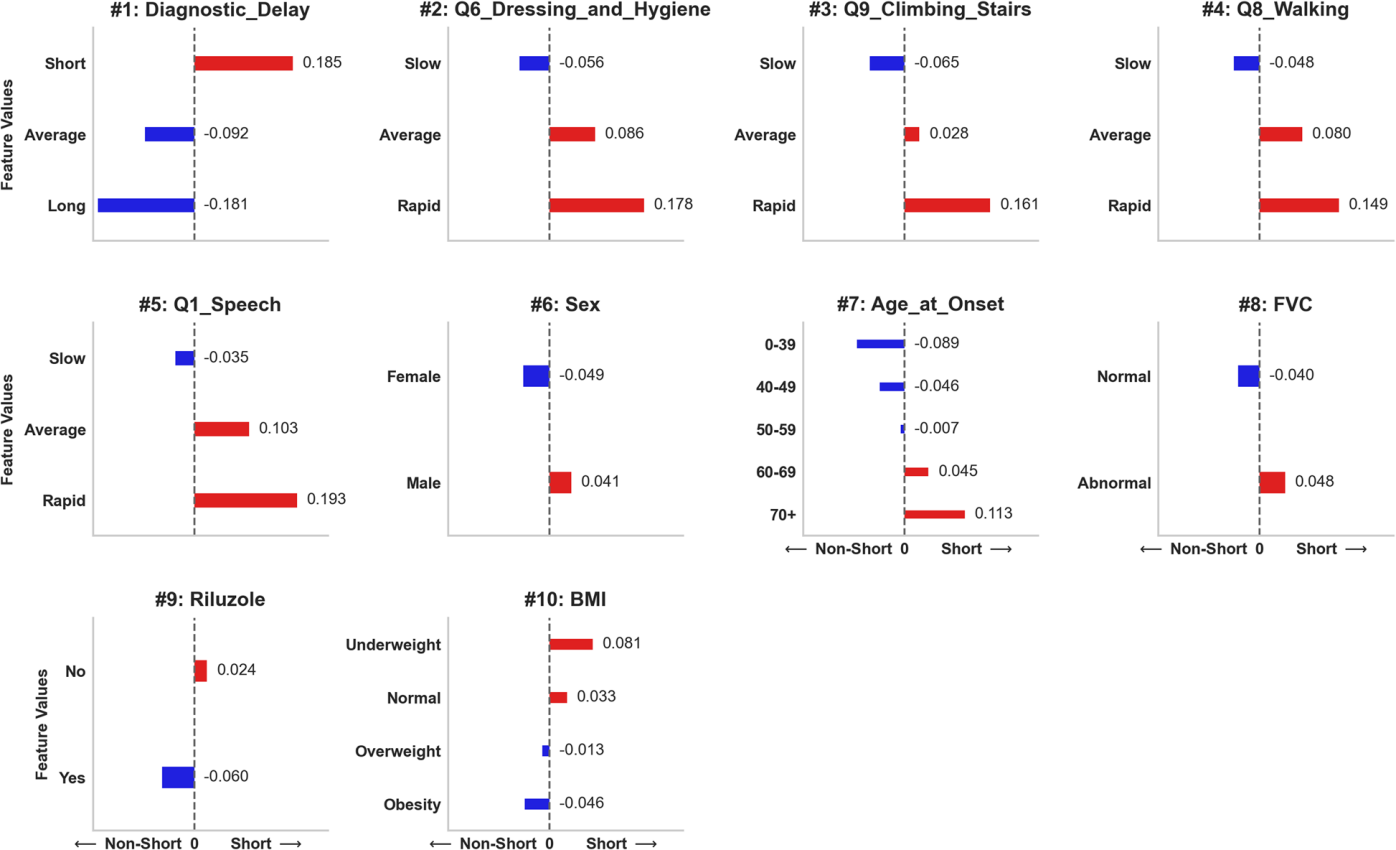
Average impact on model prediction for the top ten most important features detailed according to their values. Positive (red) and negative (blue) SHAP values drive the prediction into Short and Non-Short survival groups, respectively

Following the evaluation and selection of the overall best model (the Ensemble-Imbalance based model using NN as a base classifier), we utilized the SHAP framework to obtain insights into how this model conducted patient classifications. Figure 4 provides valuable information for comprehending the global interpretability of the model. The left graph displays the ranking of feature importance based on their average impact on the model’s output. The right graph illustrates the correlations of feature values with the target prediction. SHAP values on the x-axis exceeding zero indicate that the feature value drove the prediction into the Short survival group, whereas those below zero into the Non-Short group. Figure 5 elucidates the global interpretability by detailing the impact on model prediction according to each feature value. Due to space constraints, we present the top ten most relevant features.

**Fig. 6:**
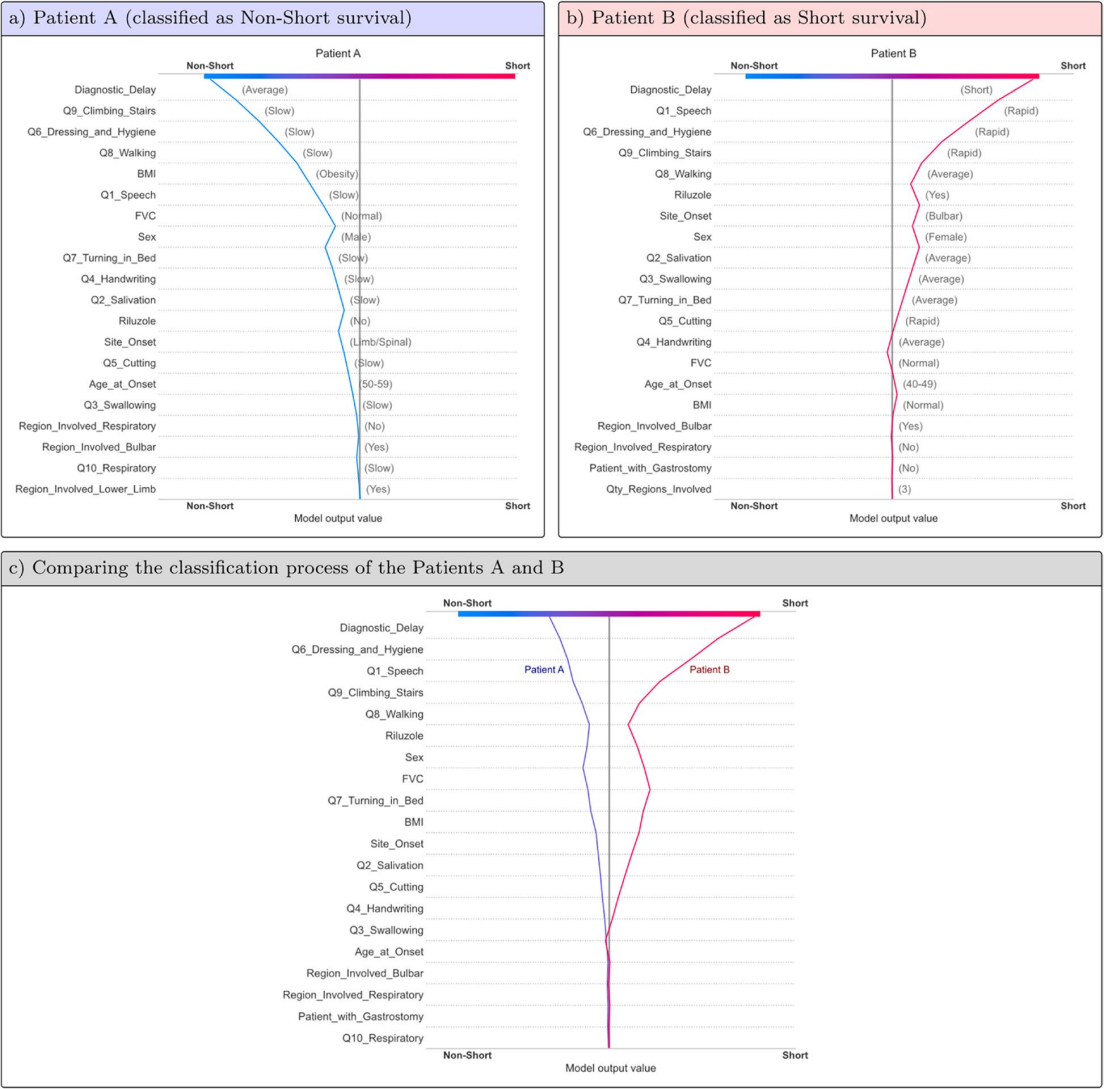
Examples of using the SHAP Decision plot to explain how the model classified patients into Short and Non-Short survival groups. Subfigures “**a**” and “**b**” show individualized classifications for each patient, where the process was conducted according to their feature values (displayed in gray font within parenthesis). Subfigure “**c**” shows the classification process comparing feature by feature for both patients. These graphs must be read from bottom to top. The slope of the line within each feature area indicates when the feature value drove the prediction toward the Non-Short (left sloping) or the Short (right sloping) groups. The longer the line length within the feature area, the more significant the impact of its value on the model prediction

Figure 6 offers an illustration of how SHAP local interpretability can be leveraged to elucidate the classification of any given patient based on their feature values. This Figure displays information for two patients (A and B) extracted from the Validation set. While Patient A was classified into the Non-Short survival group, Patient B was placed into the Short group. Subfigures “a” and “b” show individualized classifications for both patients. The classification process was driven differently according to their feature values (displayed in gray font within parenthesis). Subfigure “c” illustrates the classification process by comparing both patients on a feature-by-feature basis.

## Discussion

This study assessed the application of Ensemble and Imbalance Learning to enhance the prediction of short-survival ALS patients at the time of diagnosis. Our focus was on the analysis of patient data commonly encountered in routine ALS clinical practice, obtained through a less complex process. We discuss the results obtained in the following subsections.

### Predictive performances

In the Ensemble-Imbalance scenario, most of the algorithms (5 out of 6) exhibited significantly improved performance when compared with the Single-Model scenario (Fig. 3). The proposed Ensemble-Imbalance approach notably increased Sensitivity without compromising Balanced Accuracy. This is crucial as it improves the classification of critical patients. The only exception was Naïve Bayes, where the difference between the scenarios was not statistically significant (*p*-value: 0.346). In the Single-Model scenario, k-NN was the most affected by the data imbalance problem, achieving a Balanced Accuracy of 0.68 and showing a tendency to favor the majority class (Non-Short).

The Ensemble-Imbalance-based model using Neural Networks as a base classifier (EI-NN) outperformed the others significantly (Balanced Accuracy: 0.88; Sensitivity: 0.96; Specificity: 0.80; *p*-value ≤ 0.001). The Decision Tree, SVM, and Random Forest models demonstrated similar performances to EI-NN. We assume these four models are proper for composing a CDS system inference mechanism for classifying critical ALS patients based on data collected at diagnosis. Our approach yielded promising results, but further validation with unseen data, preferably real-world patient data, is necessary to eliminate bias toward the minority class (Short). This step is essential for a more robust model comparison.

### Data preprocessing

The data preprocessing proposed and executed in this study proved to be highly efficient, enabling ML algorithms to gain a comprehensive understanding of ALS characteristics. Even in the Single-Model scenario, Neural Networks, Random Forest, and SVM models achieved good performance, considering the data imbalance and the complexity of ALS. In the context of ALS prognosis, a data categorization approach may be more effective than direct utilization of the actual feature values. Future studies could explore alternative definitions of categorical values to assess their impact on performance.

Our results also highlighted the feasibility of constructing ML solutions using less complex biomarkers. We consider it essential to develop feasible CDS systems for primary care, eliminating the need for more complex and costly biomarkers such as genetics.

ALS disease presents an inherent complexity, and future work can extend our approach by employing clustering methods to find groups of patients with correlated clinical features and, thus, develop more effective models based on the clusters identified. We can cite FCAN-MOPSO [23] and Biclustering [24] as examples of such methods.

### Features importance and model explanation

A comprehensive analysis of the results revealed valuable insights into understanding the global interpretability of the model, the importance of the features, and their correlations with target prediction (refer to Figs. 4 and 5). Many features displayed substantial correlations with the target, underscoring their importance in identifying critical patients at the time of diagnosis. Table 2 provides details on the type of correlation (positive/negative) for the top ten ranked features based on their categorical-ordinal values. Diagnostic Delay, BMI, and Riluzole exhibited the most relevant negative correlations with the target. Conversely, Q6-Dressing & Hygiene, Q9-Climbing Stairs, Q8-Walking, Q1-Speech, Sex, Age at Onset, and FVC showed the most relevant positive correlations.

**Table 2:** Features correlations with the target variable ordered by the type and importance

Correlation Type	Feature	Ordering of the Categorical Values
Negative	Diagnostic Delay	Short → Average → Long
	Riluzole	No → Yes
	BMI	Underweight → Normal → Overweight → Obesity
Positive	Q6 − Dressing & Hygiene	Slow → Average → Rapid
	Q9 − Climbing Stairs	
	Q8 − Walking	
	Q1 − Speech	
	Sex	Female → Male
	Age at Onset	[0 − 39] → [40 − 49] → [50 − 59] → [60 − 69] → [70 +]
	FVC	Normal → Abnormal

The *Diagnostic Delay* was the most relevant among the features. We can observe that 66% of patients in the Short survival group were diagnosed within the first eight months of the onset of the disease (Table 1). This is an important biomarker, although it is necessary to analyze the following features to understand which signs led to a faster diagnosis. Following the ranking, questions *Q6*, *Q9*, and *Q8* of the ALSFRS scale appear as the most relevant, thus correlating the degree of lower motor neuron degeneration with a worse prognosis. This aligns with what was reported by Al-Chalabi et al. [25]. Other factors that represented a worse prognosis also conform with the literature on ALS, such as male gender, being older at diagnosis, and having an abnormal FVC. Previous studies using ML applied to ALS prognosis have also identified these features as survival predictors [6, 15, 16, 26]. Hence, we conclude that the proposed Ensemble-Imbalance approach effectively learned from patient data to extract crucial ALS disease characteristics.

The most significant characteristics for identifying critical ALS patients at the time of diagnosis were: (i) shorter diagnostic time (≤ 8 months); (ii) higher decline (*slope* ≥ 0.14) in ALSFRS Q6 (Dressing & Hygiene), Q9 (Climbing-Stairs), Q8 (Walking), and Q1 (Speech); (iii) male gender; (iv) age ≥ 60 years old; (v) abnormal FVC; (vi) not treated with Riluzole; and (vii) underweight (BMI ≤ 18*.*4).

Figure 6 illustrates the local interpretability of the model based on the SHAP results. Subfigures “a” and “b” provide personalized predictions for two patients extracted from the Validation set. Patient A was classified into the Non-Short survival group, whereas Patient B was classified into the Short. Please note that their feature values influenced the classification process (displayed in gray font within parentheses). Our approach enables the identification of the most influential features contributing to disease progression. Consequently, physicians can direct symptomatic treatment to enhance the patient’s quality of life. For example, Patient B exhibited a significant functional decline in *Q1-Speech* and *Q9-Climbing Stairs*. This information could guide physicians in deciding that speech and physical therapies are necessary. Moreover, this information may be employed as inclusion or exclusion criteria in clinical trials, facilitating the selection of patients with predefined characteristics. Subfigure “c” details both patients feature-by-feature within the same graph, providing a valuable resource for visualizing and comparing two or more patients, thereby revealing their similarities and differences.

This study has certain limitations. The data analyzed were extracted from clinical trials rather than a population-based registry. Consequently, there is a risk that it may not fully represent the entire ALS population due to the inclusion and exclusion criteria applied. Furthermore, the dataset exclusively comprised ALS patients from the United States of America. It is essential to validate the results using data from other regions with different genetic backgrounds (e.g., South America, Africa, or Asia). Additionally, information about cognitive impairment at the time of diagnosis was absent in the PRO-ACT database, likely due to its use as an exclusion criterion in clinical trials. Nonetheless, this biomarker has been previously identified as a significant contributor to a worse prognosis, independent of specific motor impairments [27]. Therefore, it potentially holds relevance as a feature for classifying critical patients at the time of diagnosis.

## Conclusion

This study evaluated the use of Machine Learning to predict short survival in ALS patients by analyzing biomarkers collected at the time of diagnosis. We focused on analyzing biomarkers commonly encountered in daily ALS clinical practice, thus avoiding the need for more complex and costly biomarkers such as genetics or imaging. Our findings demonstrate that the proposed Ensemble-Imbalance approach can significantly enhance predictive performance in classifying critical patients during diagnosis. Furthermore, we provided detailed insights into how the model generates predictions, emphasizing both global and local interpretability. In future work, we intend to leverage these findings to develop a Clinical Decision Support (CDS) system for classifying critical ALS patients using data collected from Brazilian patients. This represents a crucial step toward confirming the results obtained in this study.

### Supplementary Information

**Supplementary Material 1.**

## Data Availability

No datasets were generated or analysed during the current study.
