# Supplementary material for: Ensemble-imbalance-based classification for amyotrophic lateral sclerosis prognostic prediction: identifying short-survival patients at diagnosis
Source: BMC Med Inform Decis Mak. 2024 Mar 19;24:80. doi: 10.1186/s12911-024-02484-5 (PMC10949816; doi:10.1186/s12911-024-02484-5)
Supplement: Supplementary file 1 — Supplementary Material 1. [file 12911_2024_2484_MOESM1_ESM.pdf]

Supplementary Information

Ensemble-Imbalance-based Classification for Amyotrophic Lateral Sclerosis Prognostic Prediction: Identifying Short-Survival Patients at Diagnosis

Fabiano Papaiz [a, b, c, *], Mario Emílio Teixeira Dourado Jr. [a], Ricardo Alexsandro de Medeiros Valentim [a], Rafael Pinto [a, c], Antônio Higor Freire de Morais [c], and Joel Perdiz Arrais [b]

[a] Federal University of Rio Grande do Norte, Natal, Brazil
[b] University of Coimbra, Coimbra, Portugal
[c] Federal Institute of Rio Grande do Norte, Natal, Brazil

Table of Contents

Input and Output Variable Distributions. ***2***

Compare the variable distributions for the 2 Survival Groups: Short and $\mathit{Non\text{-}Short}$. ***10***

Compare the output variable distribution for the $\mathit{Training}$ and $\mathit{Validation}$ subsets used to train and validate the machine learning models ***18***

Grid-Search hyperparameters used for each algorithm. ***19***

Best models hyperparameters. ***22***

Input and Output Variable Distributions.

Column Sex

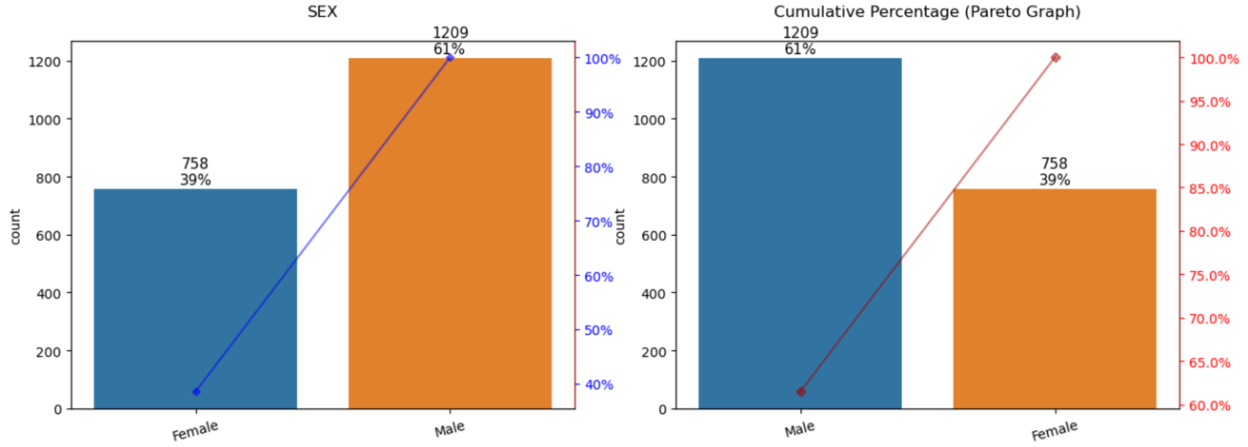

Column Site_Onset

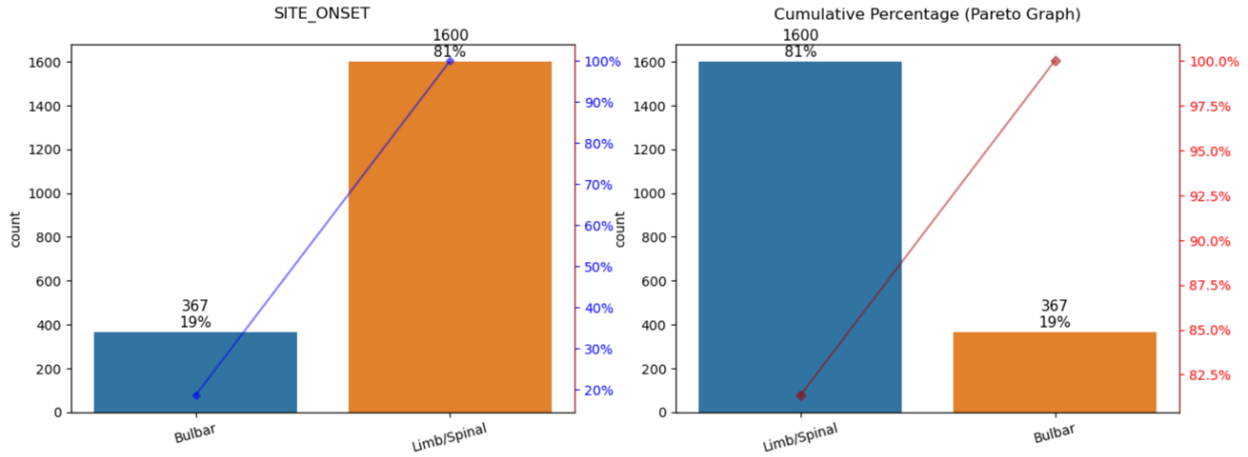

Column Diagnosis_Delay

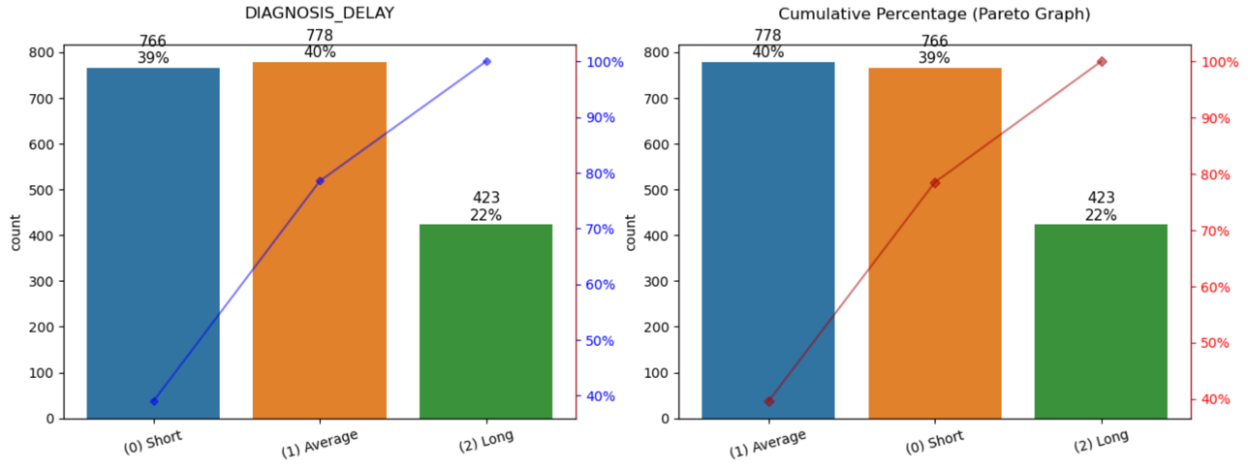

Column Age_at_Onset

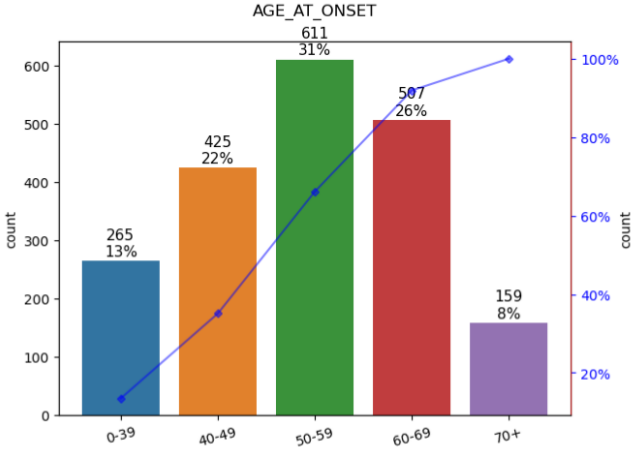

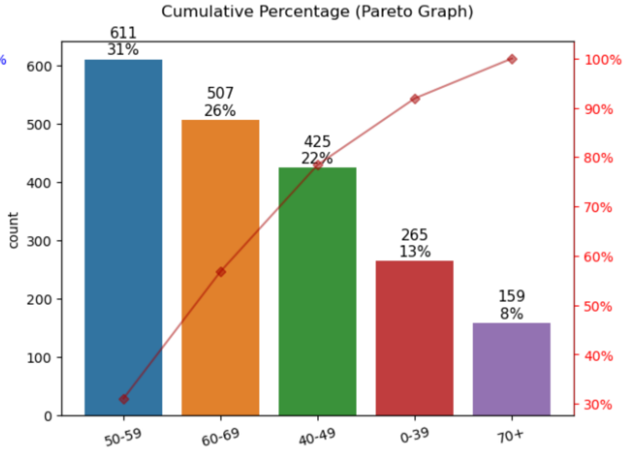

Column Riluzole

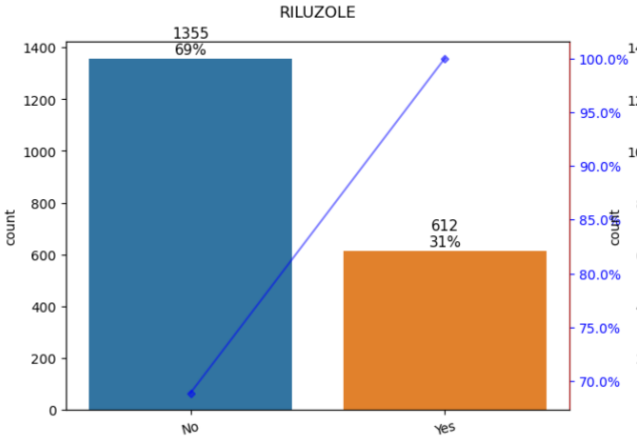

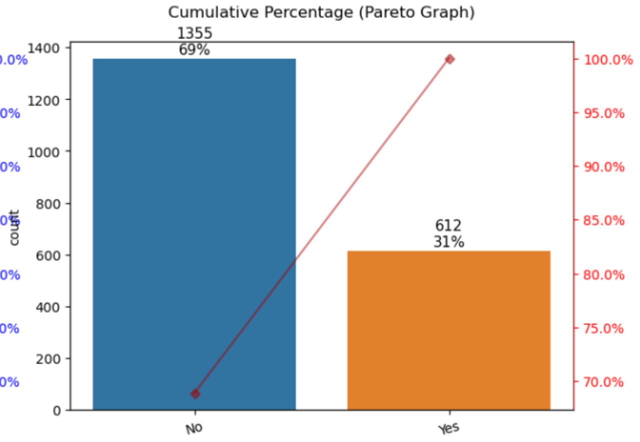

Column FVC_at_Diagnosis

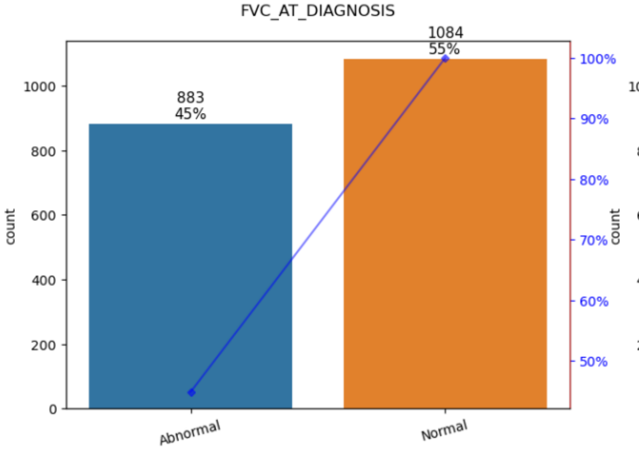

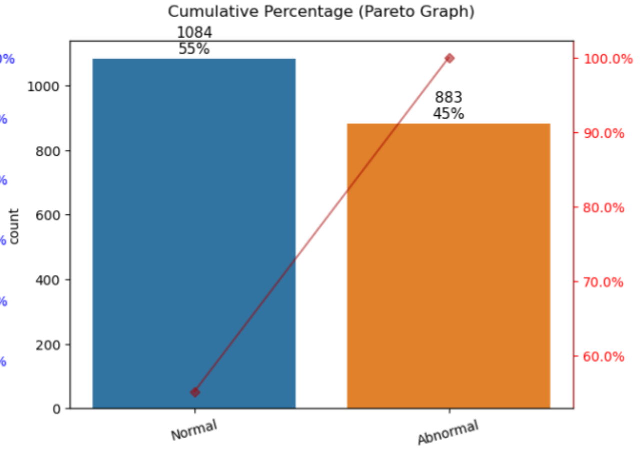

Column BMI_at_Diagnosis

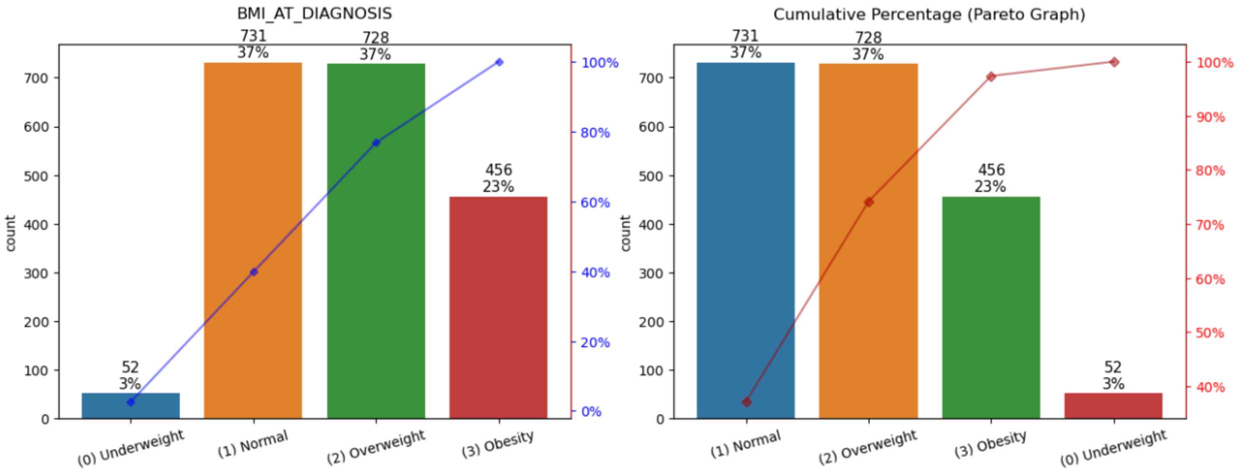

Column Q1_Speech_slope_at_Diagnosis

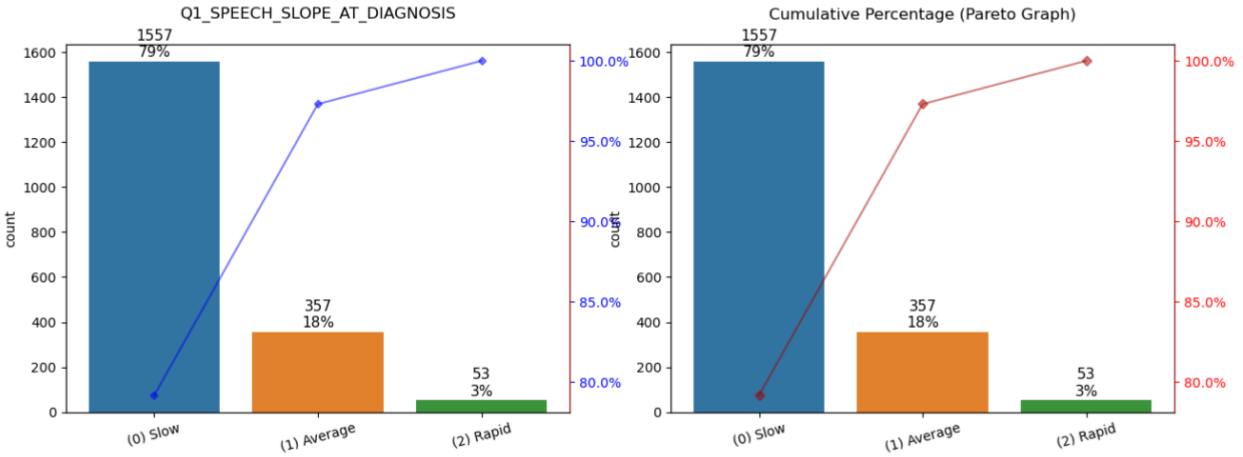

Column Q2_Salivation_slope_at_Diagnosis

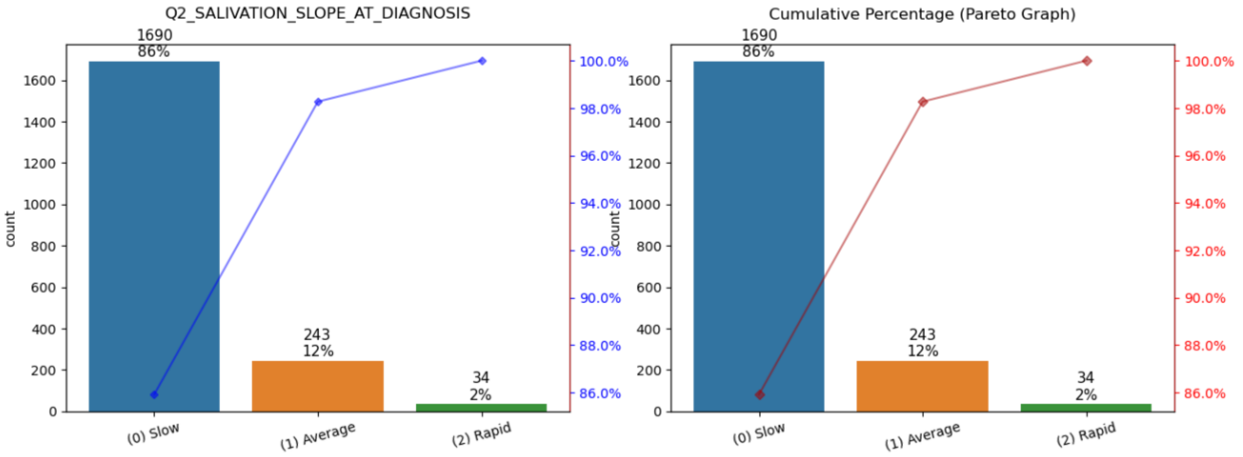

Column Q3_Swallowing_slope_at_Diagnosis

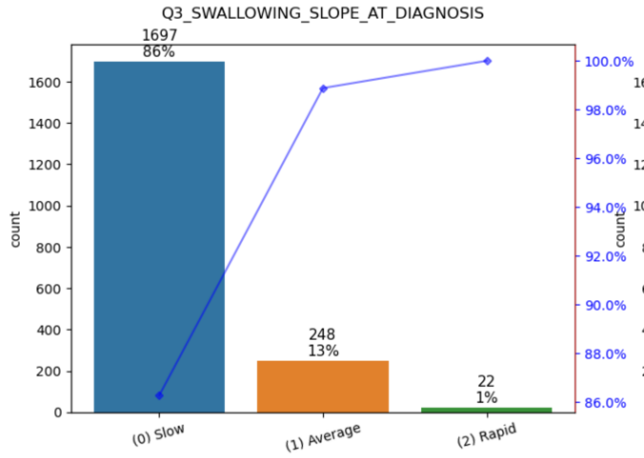

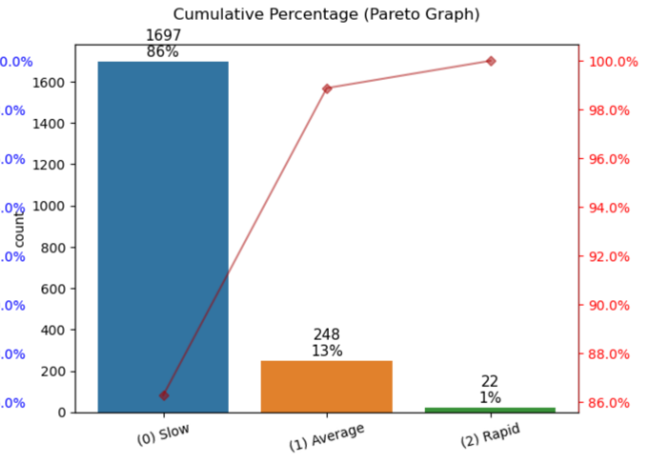

Column Q4_Handwriting_slope_at_Diagnosis

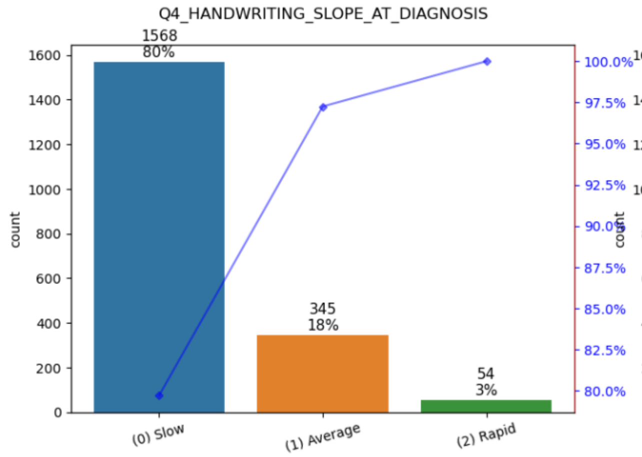

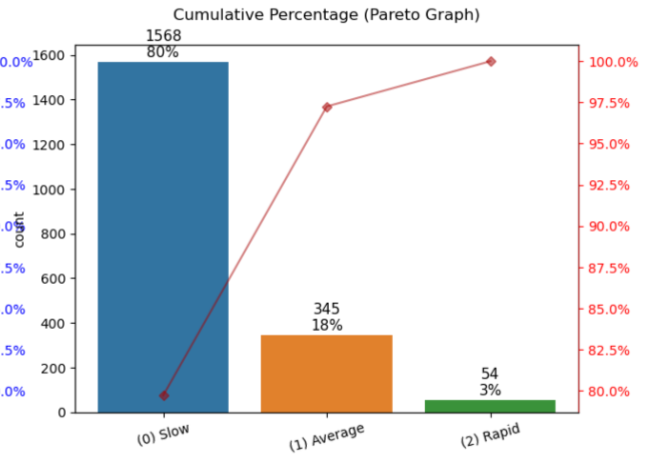

Column Q5_Cutting_slope_at_Diagnosis

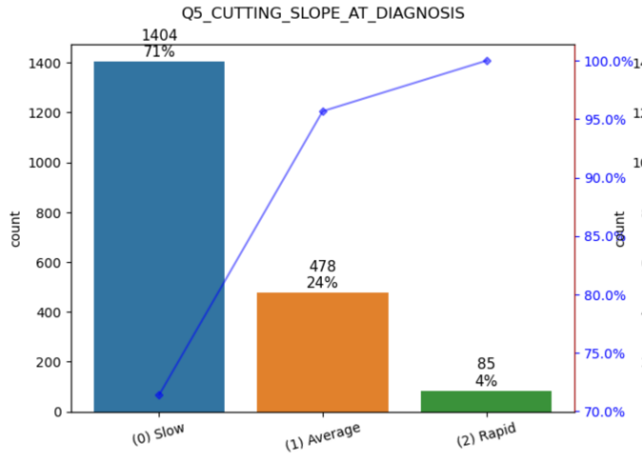

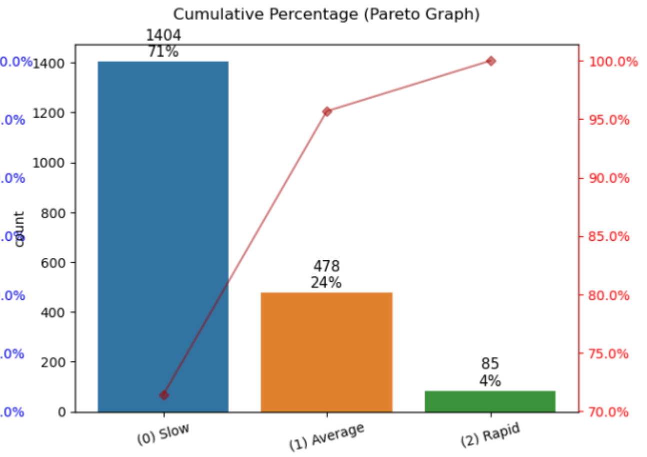

Column Q6_Dressing_and_Hygiene_slope_at_Diagnosis

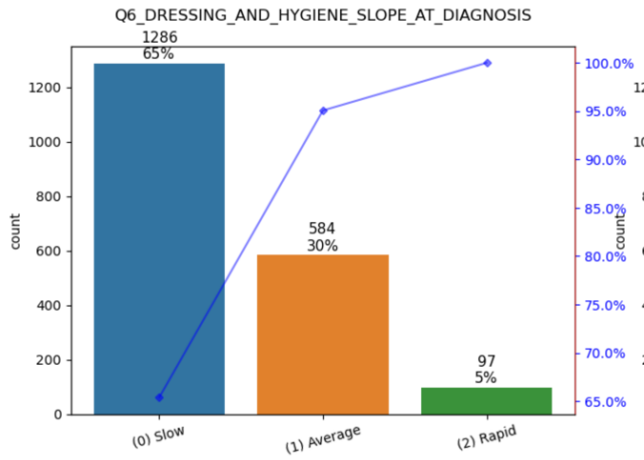

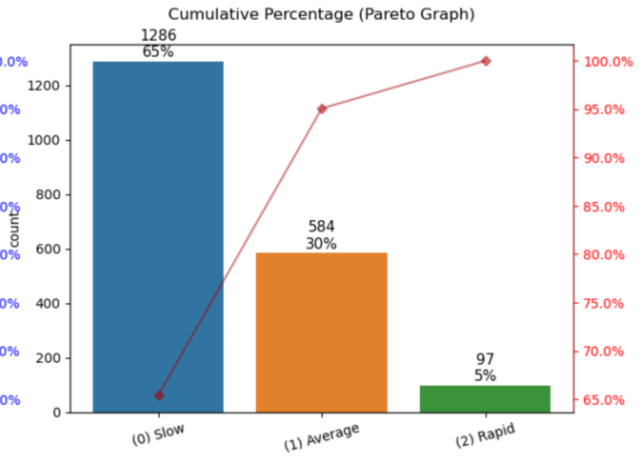

Column Q7_Turning_in_Bed_slope_at_Diagnosis

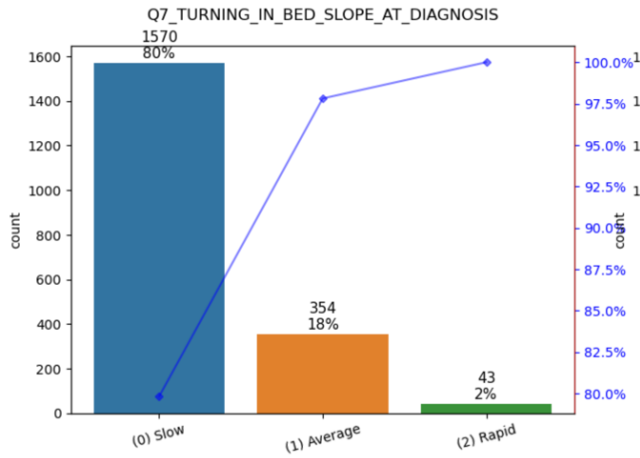

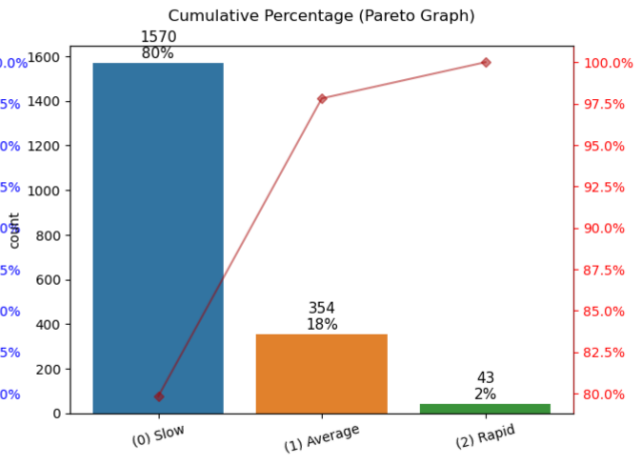

Column Q8_Walking_slope_at_Diagnosis

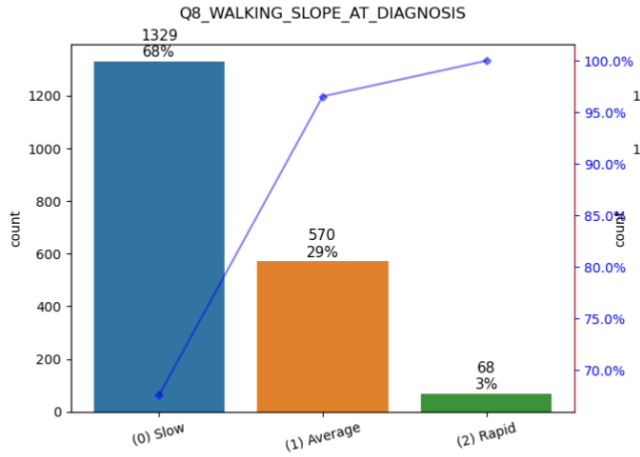

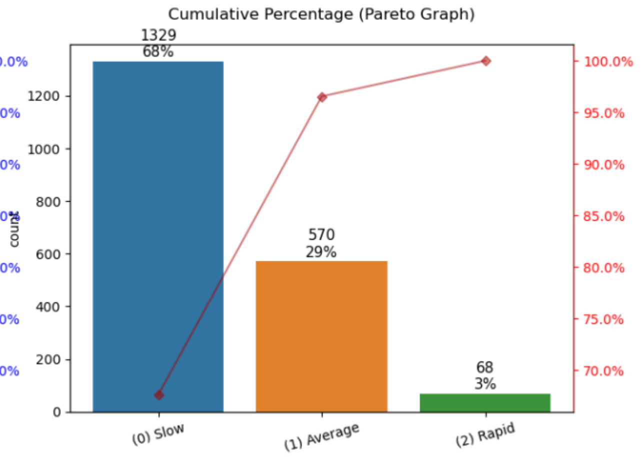

Column Q9_Climbing_Stairs_slope_at_Diagnosis

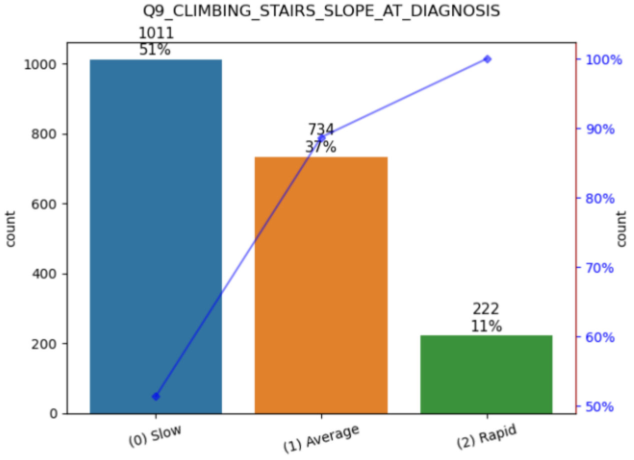

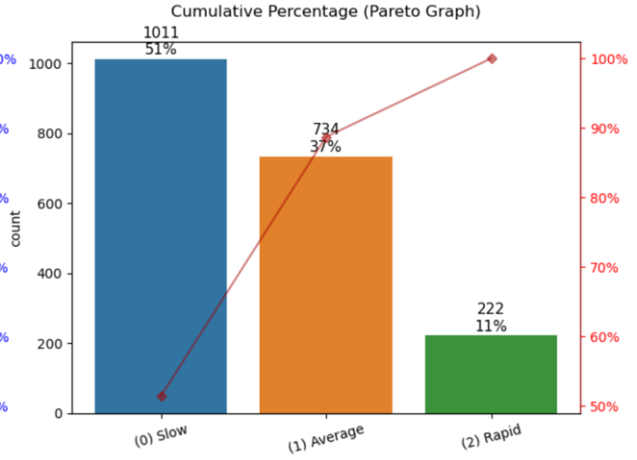

Column Q10_Respiratory_slope_at_Diagnosis

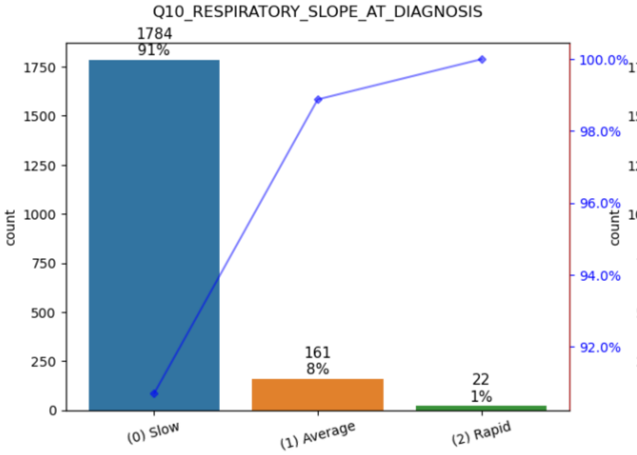

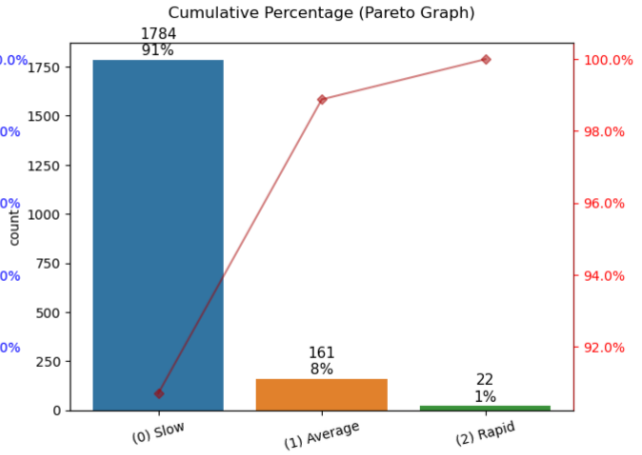

Column Qty_Regions_Involved_at_Diagnosis

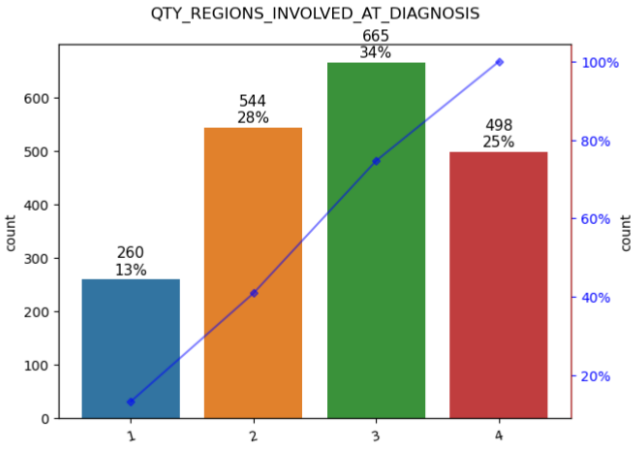

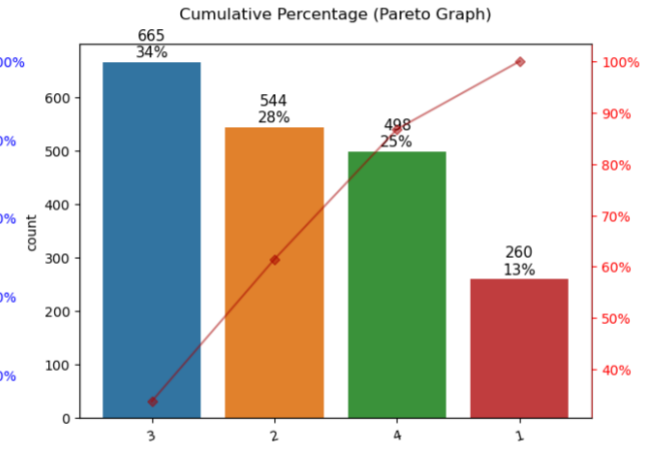

Column Region_Involved_Bulbar_at_Diagnosis

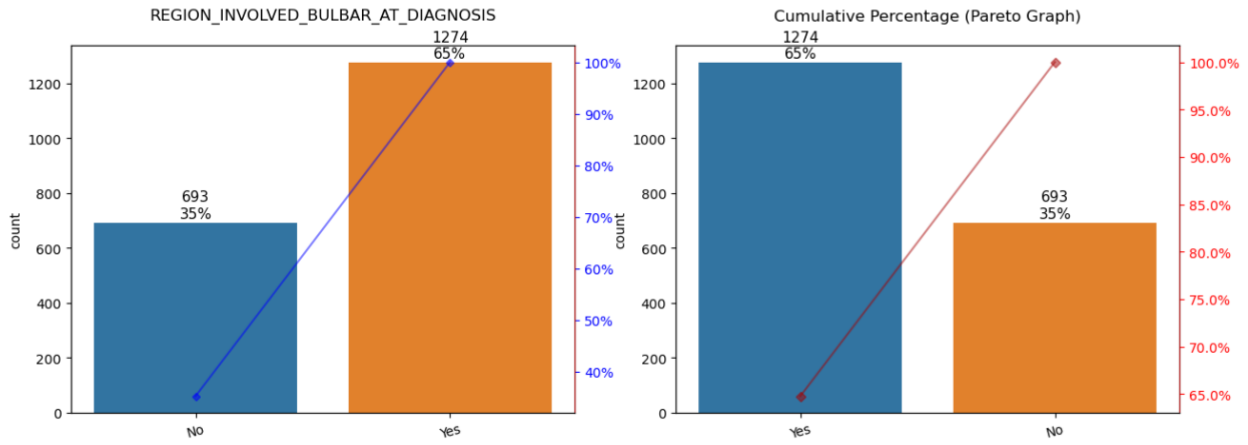

Column Region_Involved_Upper_Limb_at_Diagnosis

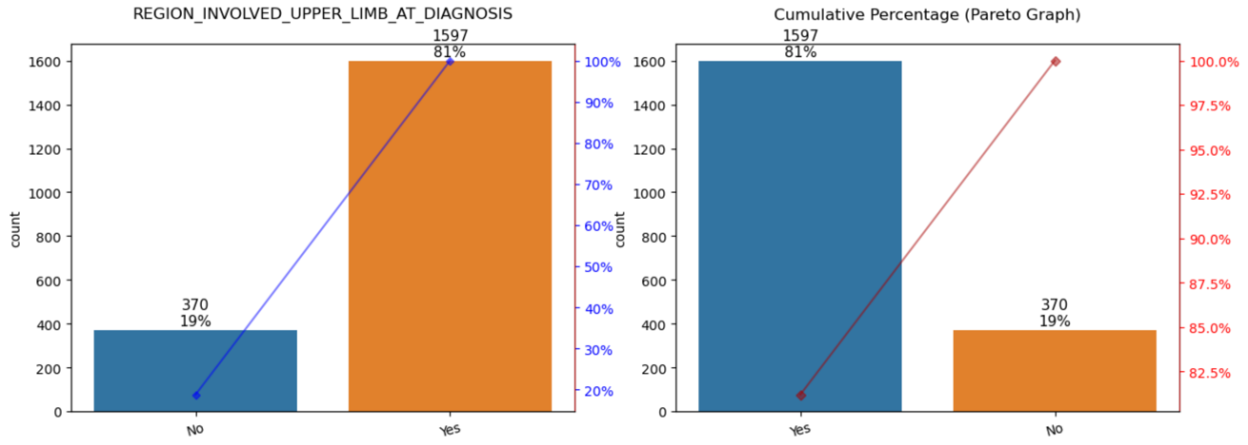

Column Region_Involved_Lower_Limb_at_Diagnosis

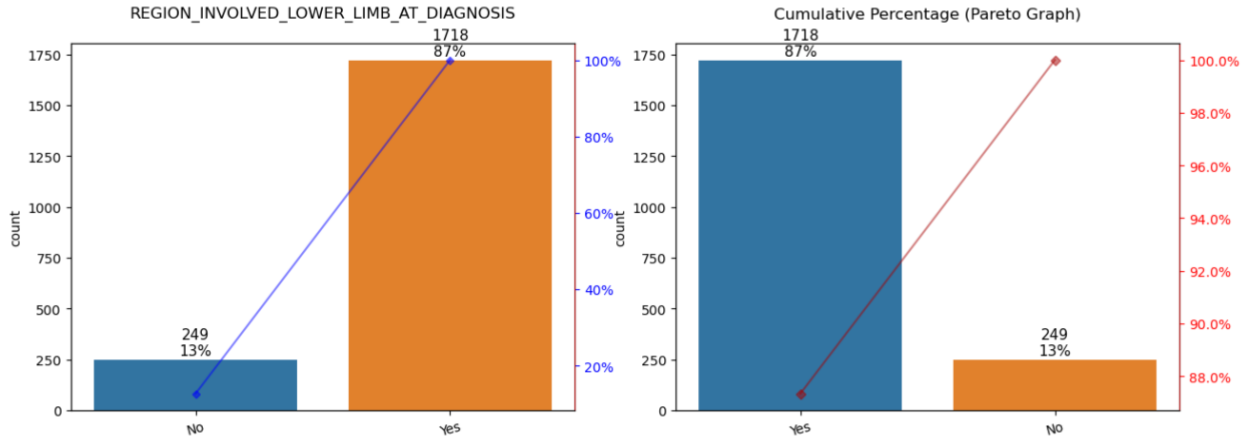

Column Region_Involved_Respiratory_at_Diagnosis

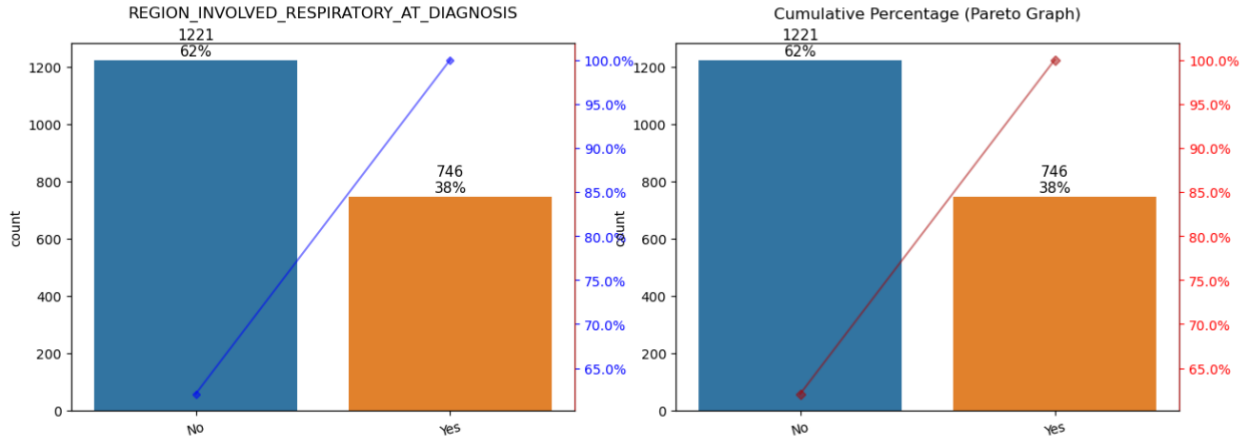

Column Patient_with_Gastrostomy_at_Diagnosis

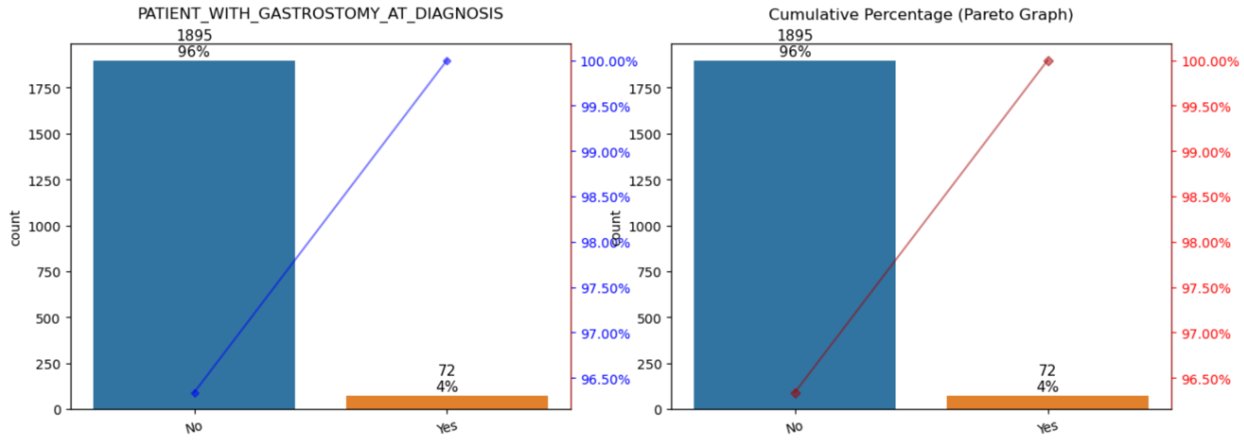

OUTPUT Column Survival_Group

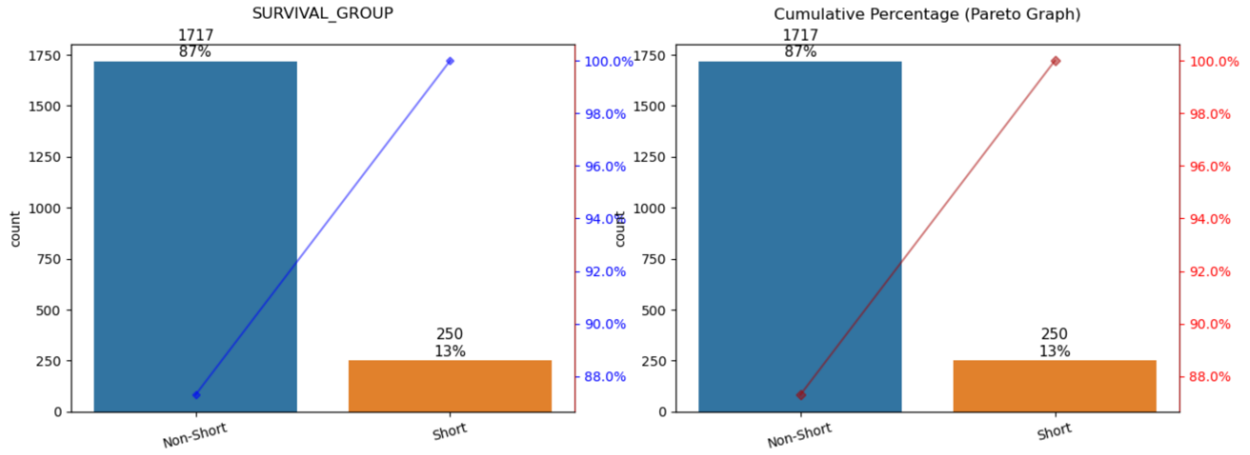

Compare the variable distributions for the 2 Survival Groups: *Short* and *Non-Short*.

`Column Sex`

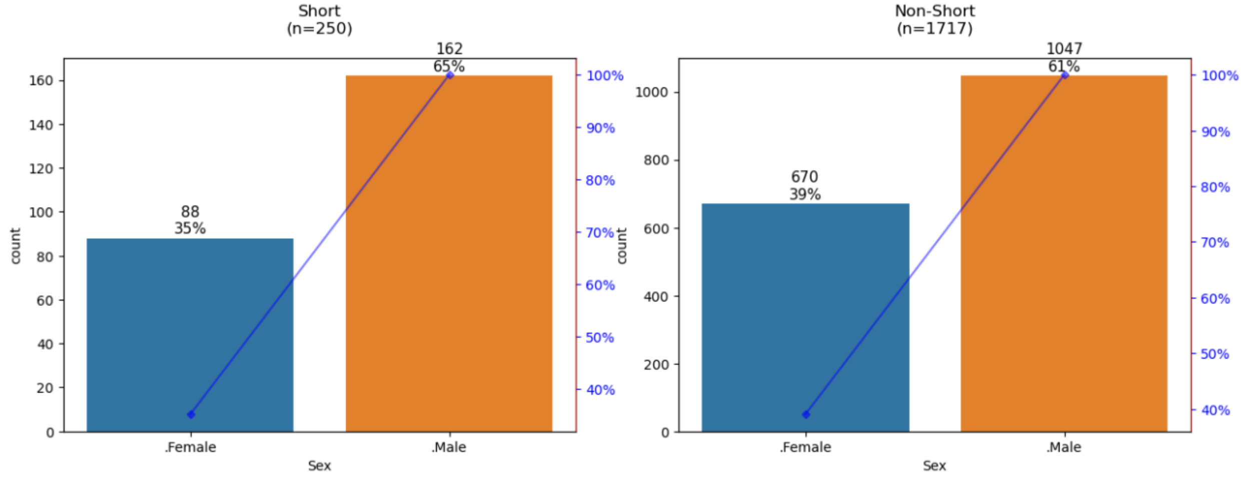

`Column Site_Onset`

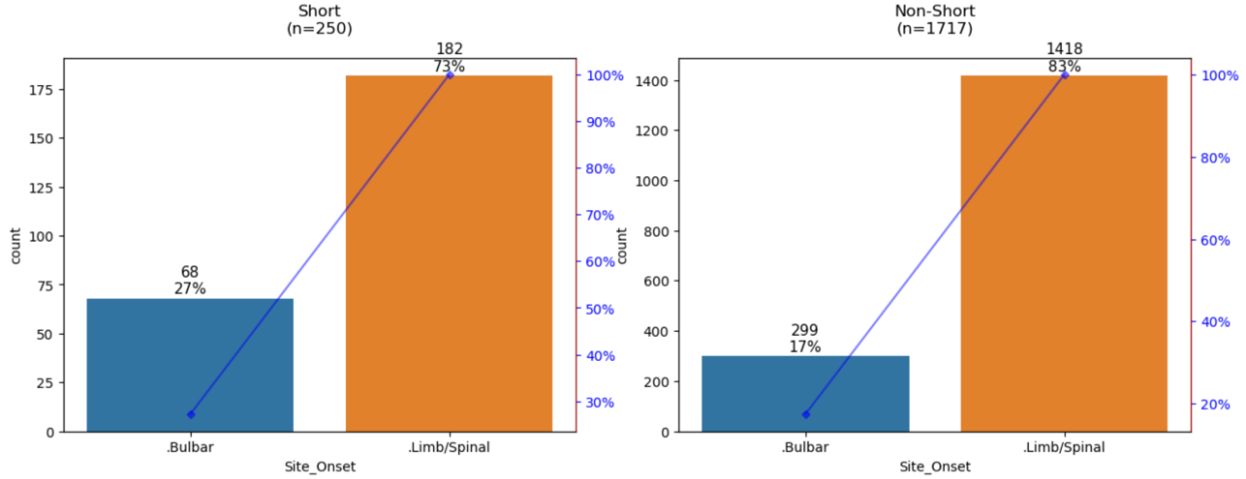

`Column Diagnosis_Delay`

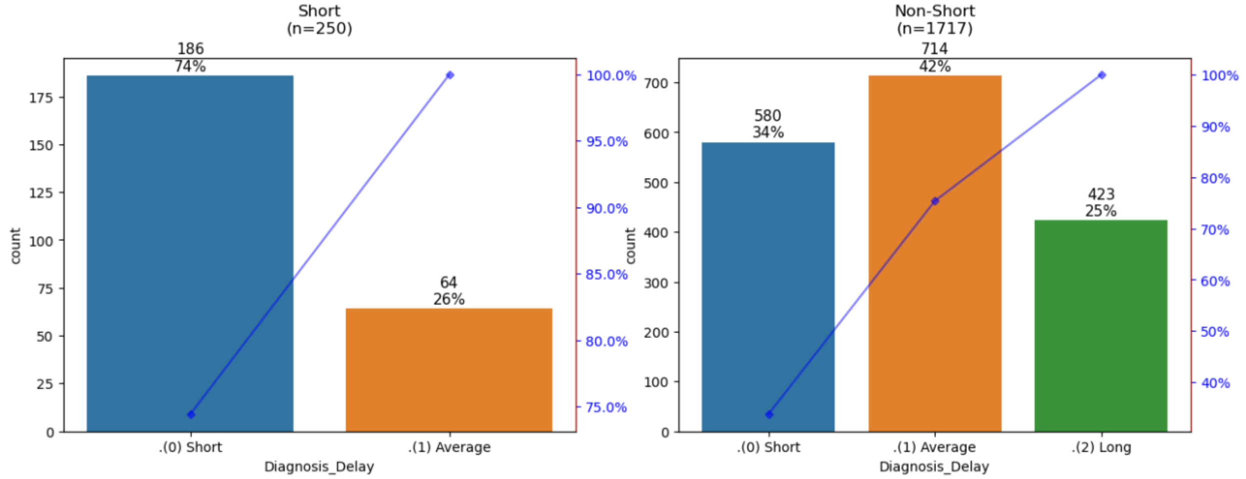

Column Age_at_Onset

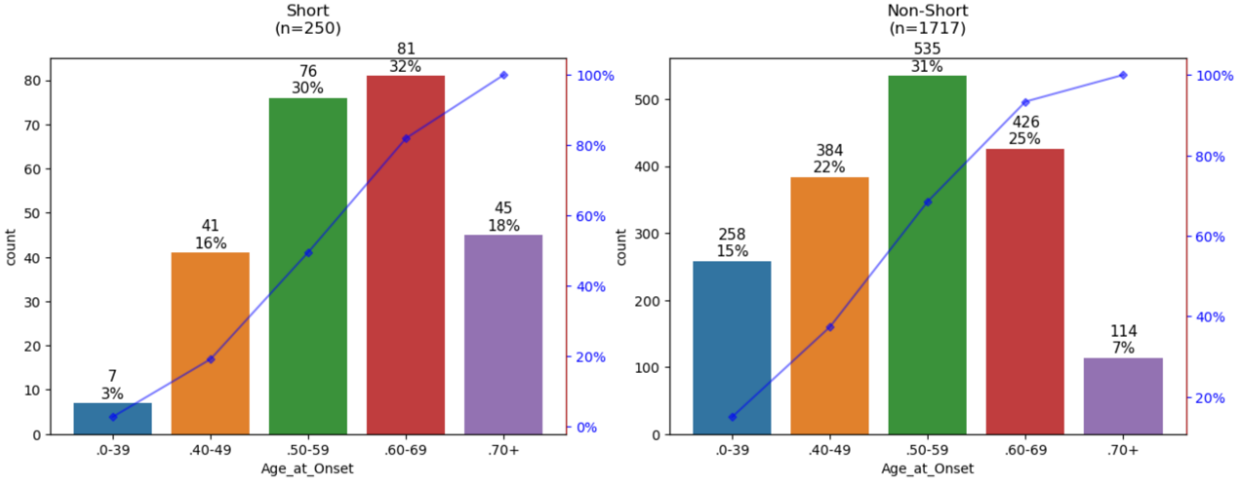

Column Riluzole

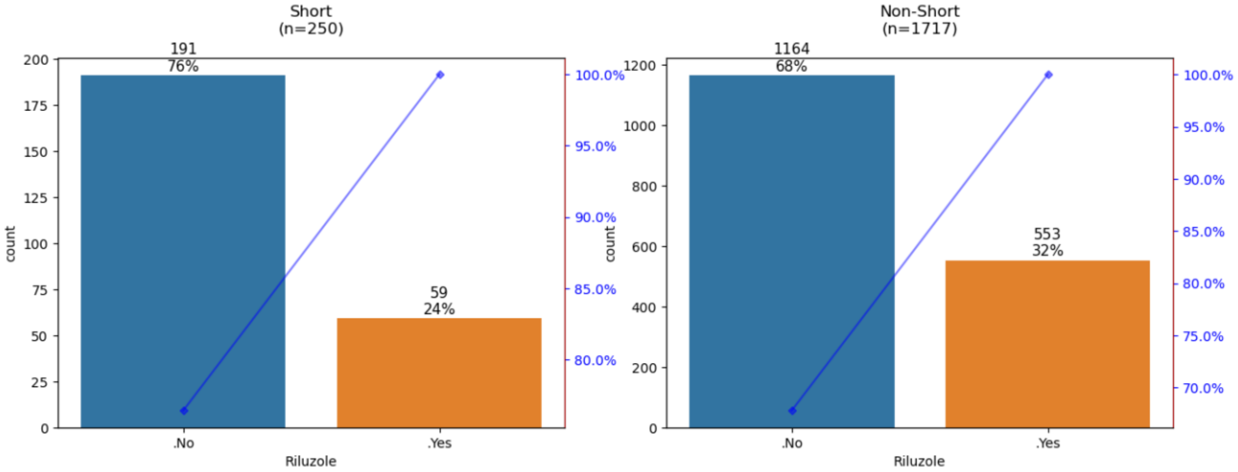

Column FVC_at_Diagnosis

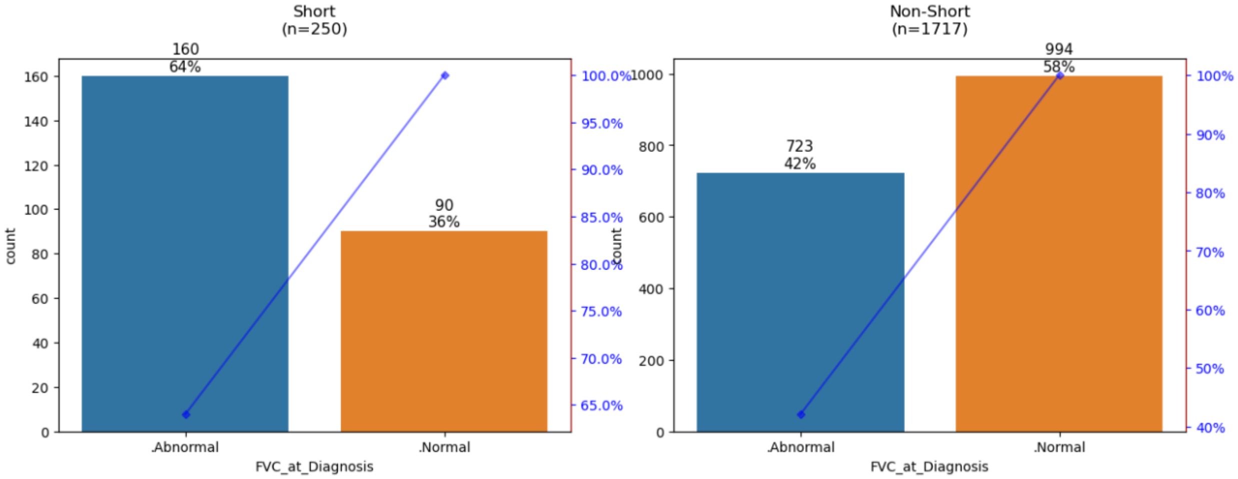

Column BMI_at_Diagnosis

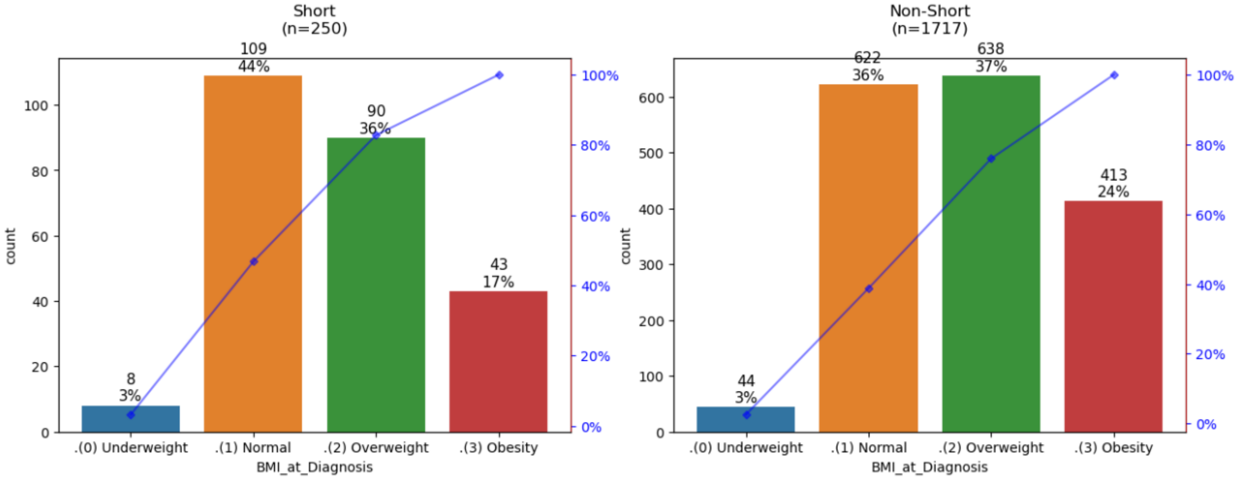

Column Q1_Speech_slope_at_Diagnosis

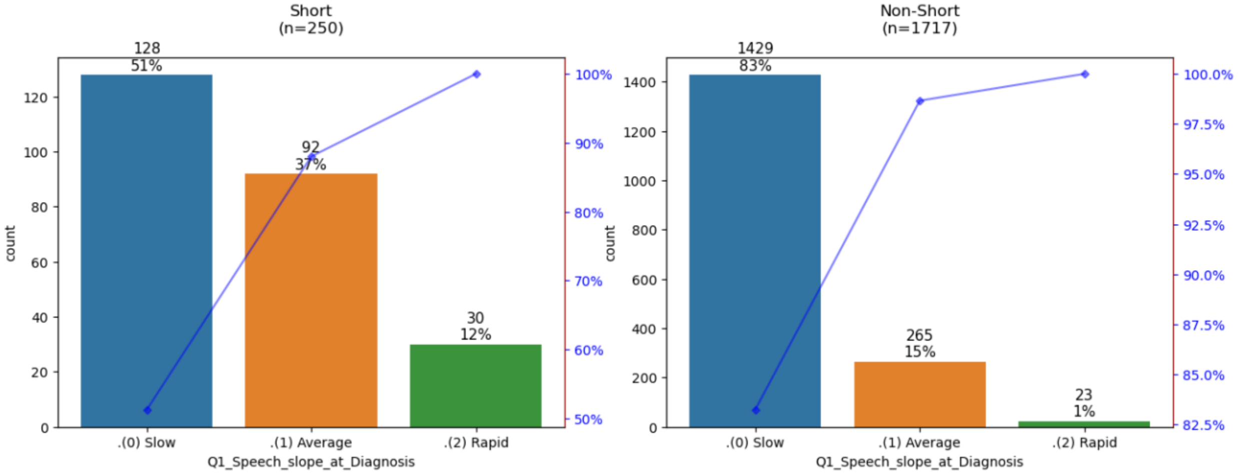

Column Q2_Salivation_slope_at_Diagnosis

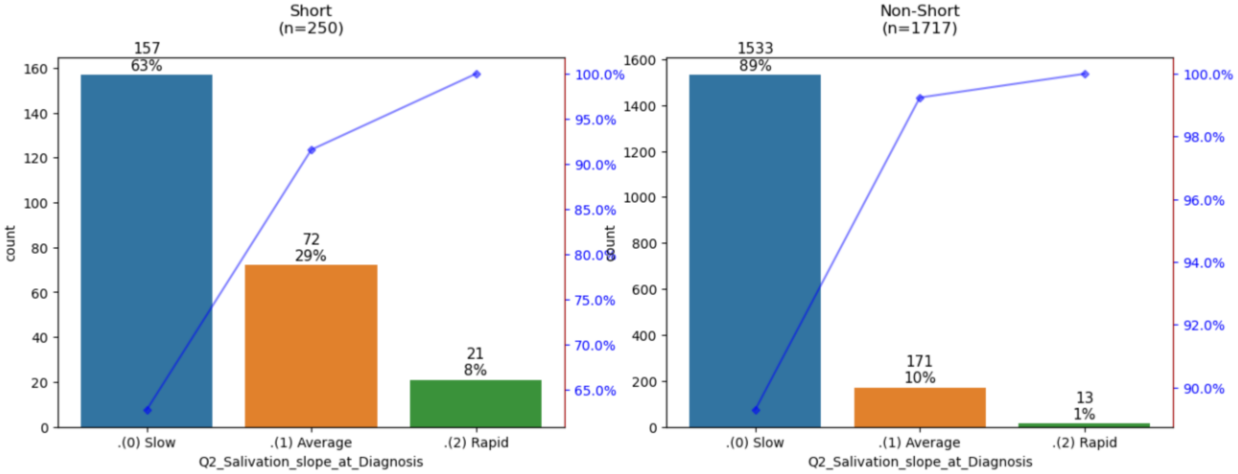

Column Q3_Swallowing_slope_at_Diagnosis

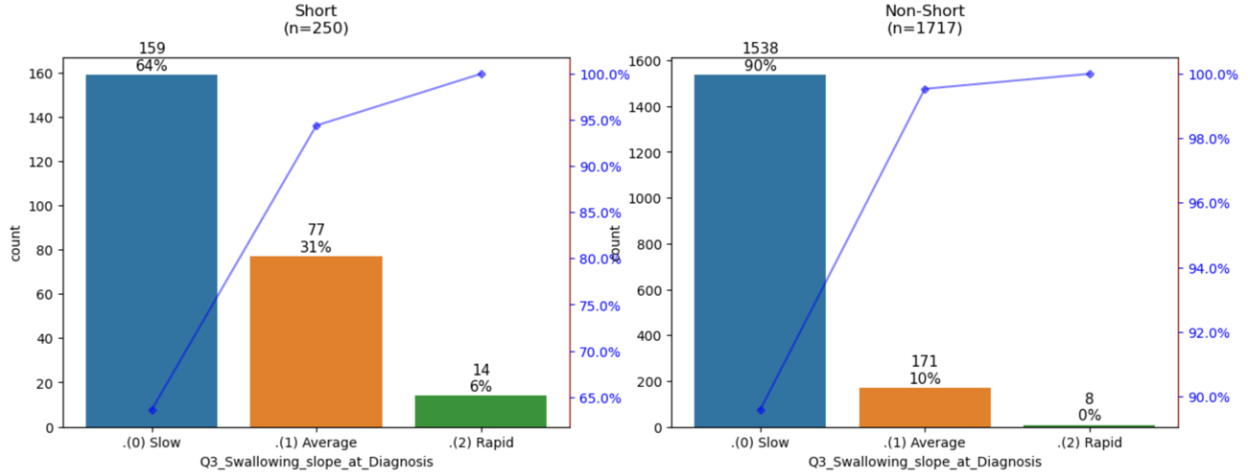

Column Q4_Handwriting_slope_at_Diagnosis

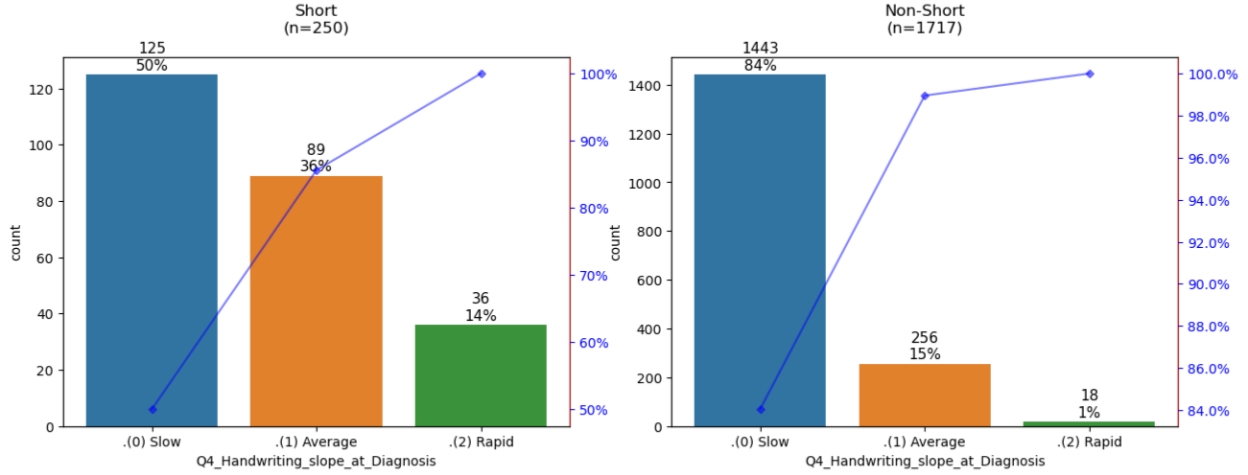

Column Q5_Cutting_slope_at_Diagnosis

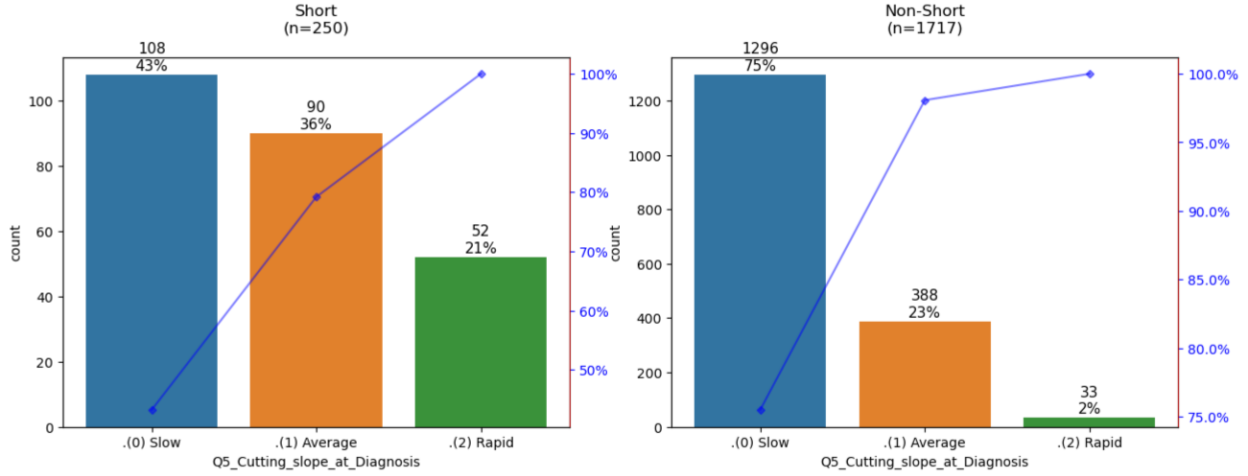

Column Q6_Dressing_and_Hygiene_slope_at_Diagnosis

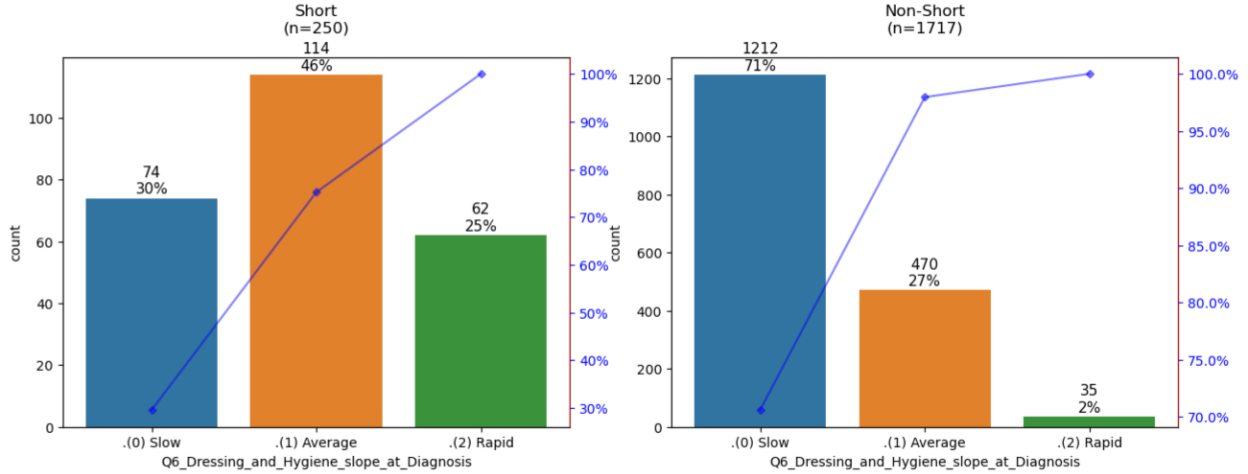

Column Q7_Turning_in_Bed_slope_at_Diagnosis

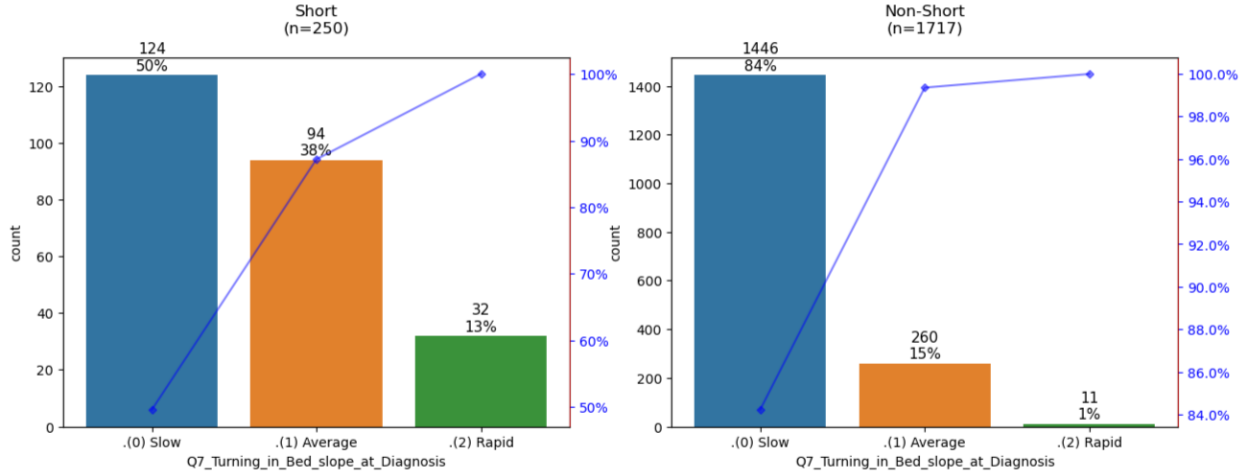

Column Q8_Walking_slope_at_Diagnosis

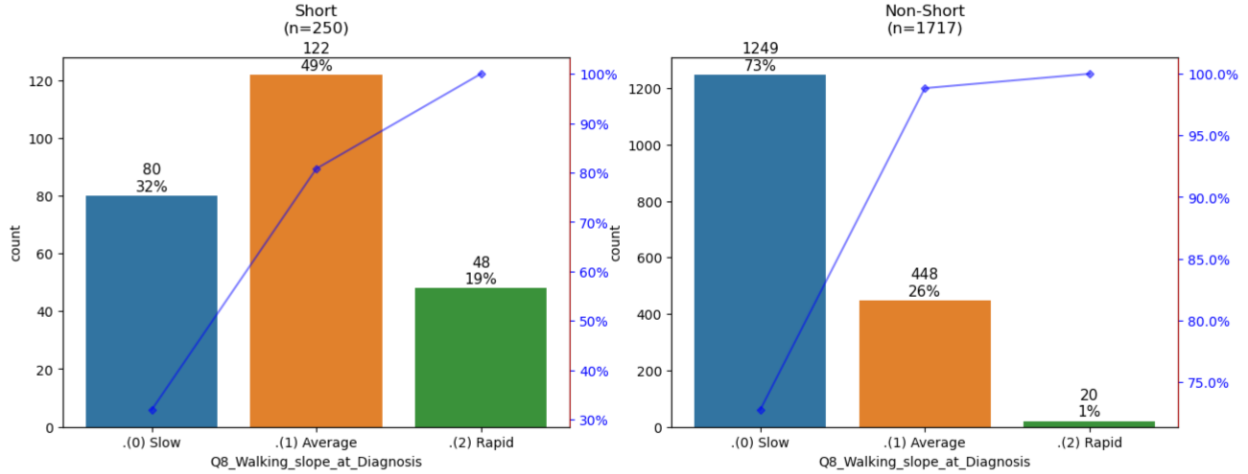

Column Q9_Climbing_Stairs_slope_at_Diagnosis

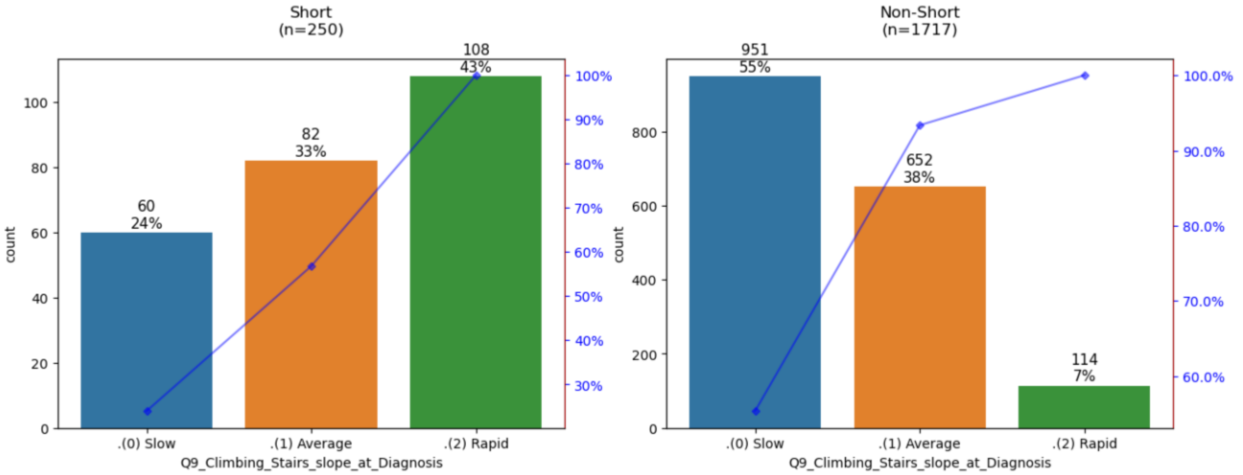

Column Q10_Respiratory_slope_at_Diagnosis

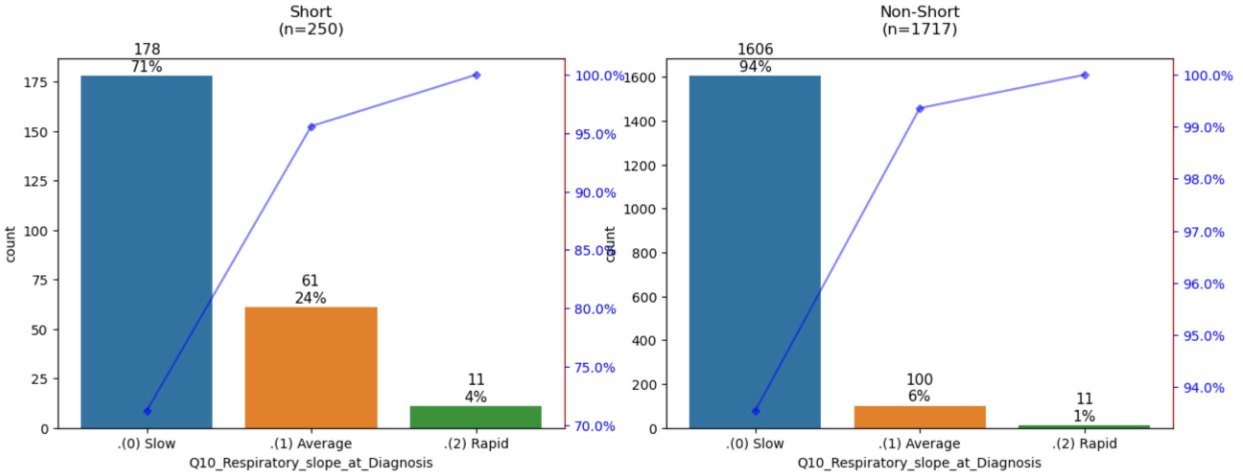

Column Qty_Regions_Involved_at_Diagnosis

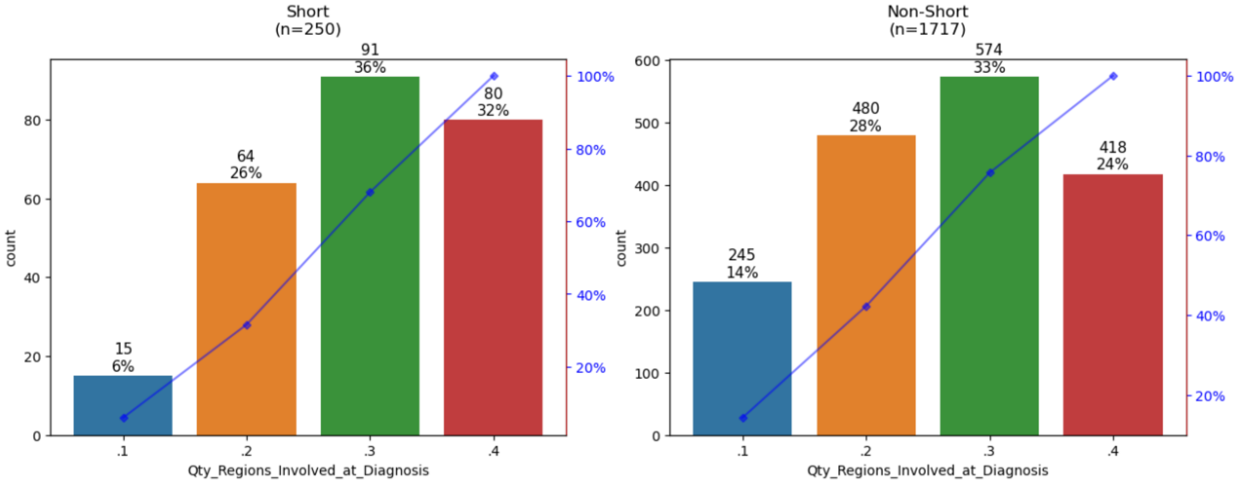

Column Region_Involved_Bulbar_at_Diagnosis

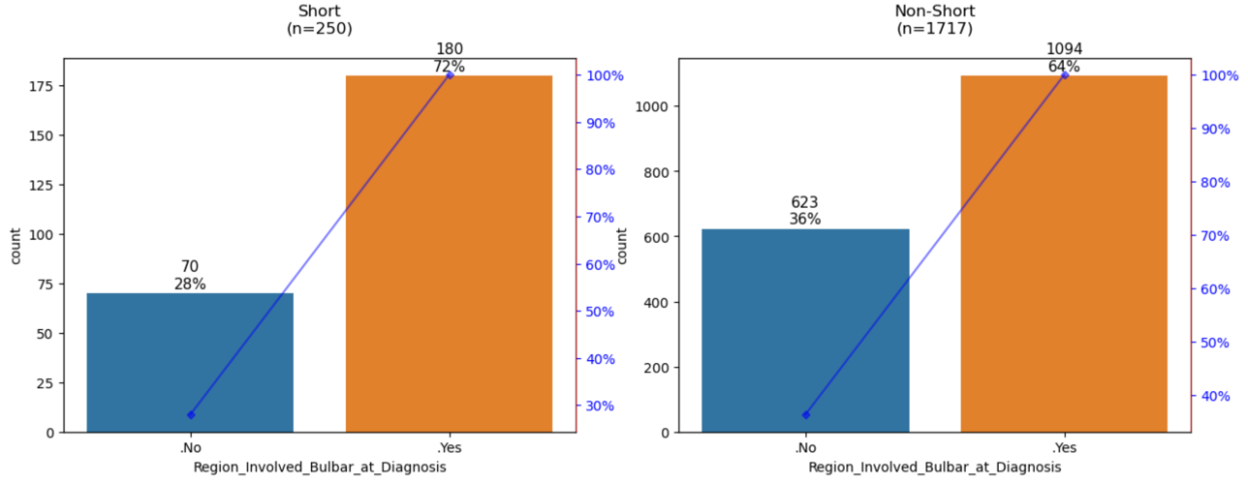

Column Region_Involved_Upper_Limb_at_Diagnosis

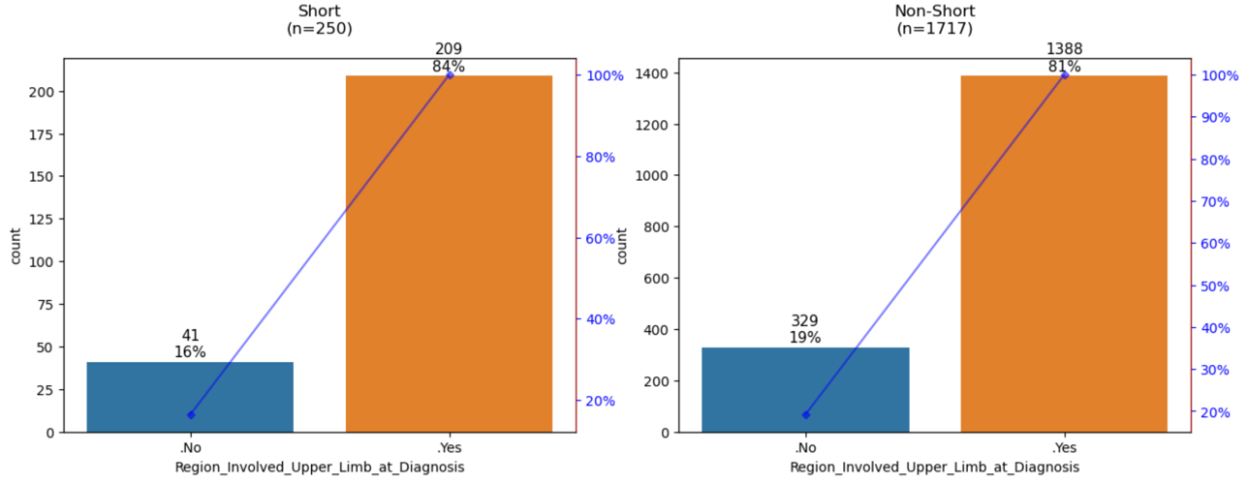

Column Region_Involved_Lower_Limb_at_Diagnosis

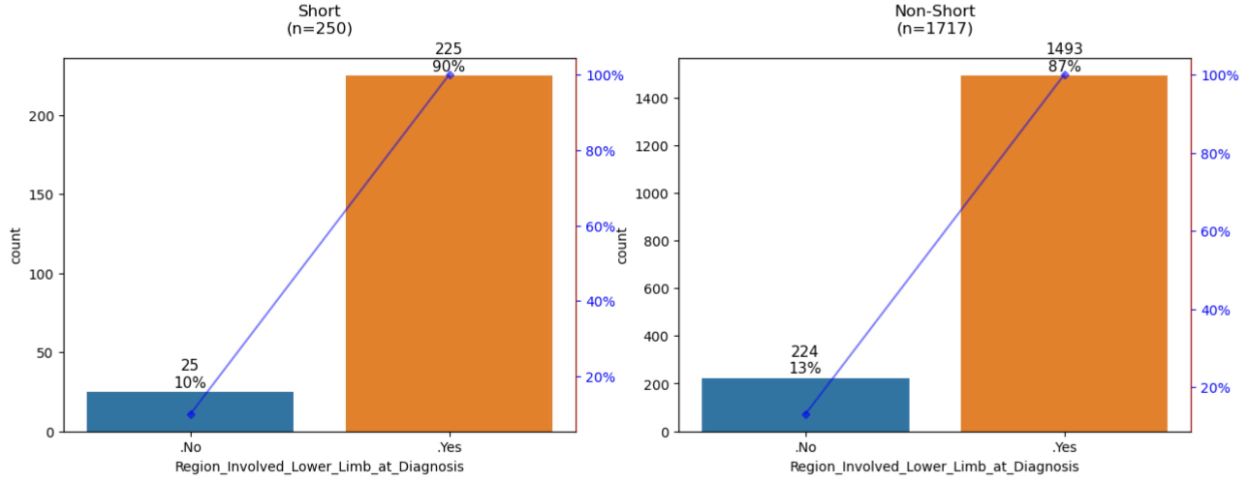

Column Region_Involved_Respiratory_at_Diagnosis

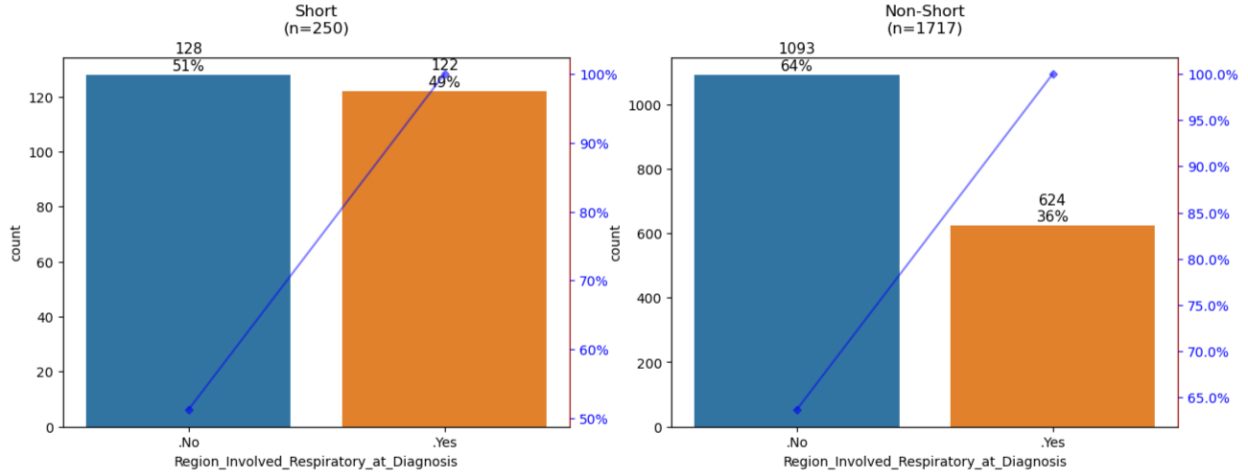

Column Patient_with_Gastrostomy_at_Diagnosis

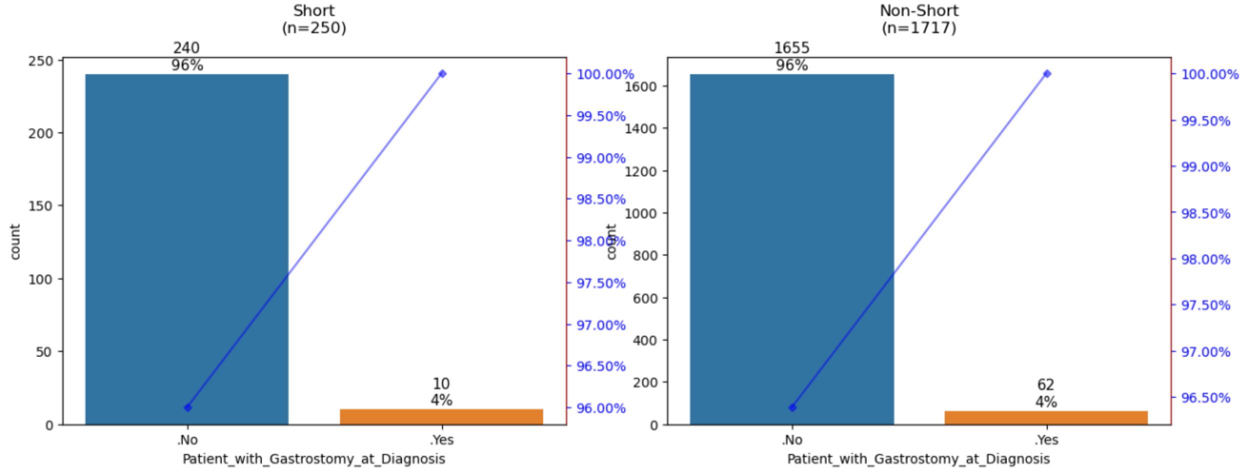

Compare the output variable distribution for the *Training* and *Validation* subsets used to train and validate the machine learning models

TRAINING

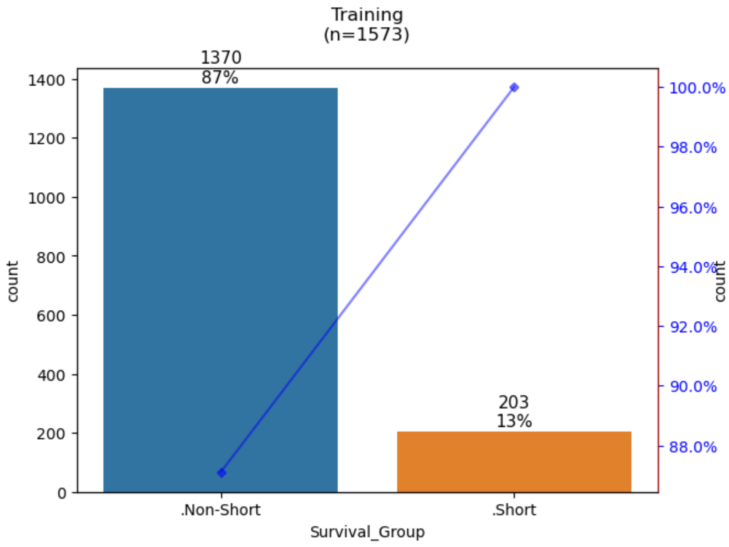

VALIDATION

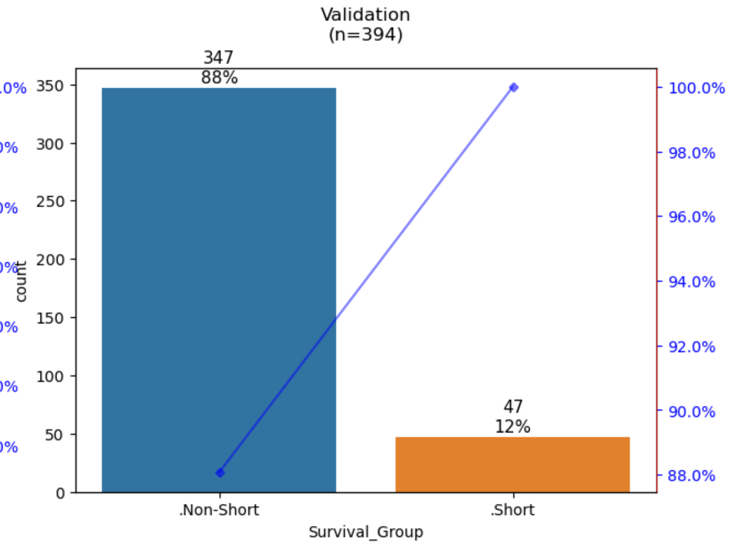

Grid-Search hyperparameters used for each algorithm.

- Decision Tree

  max_depths = [3, 4, 5, 7, 9, 10, 15, 25, 50]

  criterions = ['gini', 'entropy']

  class_weights = [None, 'balanced']

- k-Nearest Neighbors

  weights = ['uniform', 'distance']

  distance_metrics = ['euclidean', 'manhattan', 'chebyshev']

  k = [3, 5, 9, 15]

- Naïve Bayes

  alphas = [0.1, 0.5, 1.0, 1.5, 2.0, 2.5, 3.0, 3.5, 4.0]

  norms = [False, True]

- Random Forest

  max_depths = [10, 15, 25, 50]

  num_estimators = [50, 75, 100, 200]

  criterions = ['gini', 'entropy']

  class_weights = [None, 'balanced', 'balanced_subsample']

- SVM

  kernels = ['rbf', 'linear']

  gammas = ['scale', 'auto',]

```
class_weights = [None, 'balanced', ]
Cs = [0.1, 0.3, 0.5, 0.7, 1, 3, 5, 10, 100, 200, 1000, 1500, 1700, 2000]
```

- Neural Networks

```
max_iter = [1000]
alphas = [0.0001, 0.00001, 0.05, 0.1, 0.3, 0.5]
activations = ['tanh', 'relu']
solvers = ['sgd', 'adam']
learning_rates = ['constant', 'adaptive']
learning_rate_init = [0.7]
layers = [
  (30),
  (30, 30),
  (30, 30, 30),
  (qty_features, ),
  (qty_features, qty_features),
  (qty_features, qty_features, qty_features),
  (qty_features, (qty_features*2)),
  (qty_features, (qty_features*2), qty_features),
  (qty_features, (qty_features*2), (qty_features*2), qty_features),
]
```

- Balanced Bagging

```
num_estimators = [11, 15, 51, 75, 101]
sampling_strategies = ['all', 'majority', 'auto']
warm_starts = [False, True]
```

- Balanced Random Forest

```
max_depths = [5,  7,  10,  15]
criterions = ['gini',  'entropy']
num_estimators = [7,  11,  15,  19,  21,  25,  31 , 51]
sampling_strategies = ['all',  'majority',  'auto']
warm_starts = [False,  True]
replacements = [False,  True]
```

Best models hyperparameters.

Scenario	Classifier	Hyperparameters
Ensemble-Imbalance	BalancedBaggingClassifier using Decision Tree	{'estimator': DecisionTreeClassifier (class_weight='balanced', max_depth=4, random_state=42), 'n_estimators':7, 'random_state':42, 'replacement':True, 'sampling_strategy':'all', 'warm_start':False}
	BalancedBaggingClassifier using Neural Networks	{'estimator': MLPClassifier(activation='tanh', alpha=0.1, hidden_layer_sizes=30, learning_rate='adaptive', learning_rate_init=0.7, max_iter=2000, random_state=42), 'n_estimators':101, 'random_state':42, 'replacement':True, 'sampling_strategy':'auto', 'warm_start':True}
	BalancedBaggingClassifier using SVM	"{'estimator': SVC (C=0.7, class_weight='balanced', gamma='auto', probability=True, random_state=42), 'n_estimators':31, 'random_state':42, 'replacement':True, 'sampling_strategy':'majority', 'warm_start':False}
	BalancedBaggingClassifier using k-NN	"{'estimator': KNeighborsClassifier (metric='euclidean', weights='distance'), 'n_estimators':101, 'random_state':42, 'replacement':True, 'sampling_strategy':'all', 'warm_start':True}
	BalancedBaggingClassifier using Naïve Bayes	"{'estimator': GaussianNB(), 'n_estimators':19, 'random_state':42, 'replacement':True, 'sampling_strategy':'all', 'warm_start':False}
	BalancedRandomForestClassifier	{'criterion':'entropy', 'max_depth':7, 'n_estimators':19, 'random_state':42, 'replacement':True, 'sampling_strategy':'auto', 'warm_start':False}
Single-Model	DecisionTreeClassifier	{'ccp_alpha':0.0, 'class_weight':'balanced', 'criterion':'gini', 'max_depth':4, 'max_features':None, 'max_leaf_nodes':None, 'min_impurity_decrease':0.0, 'min_samples_leaf':1, 'min_samples_split':2, 'min_weight_fraction_leaf':0.0, 'random_state':42, 'splitter':'best'}
	MLPClassifier	{'activation':'tanh', 'alpha':0.3, 'hidden_layer_sizes':(23,23,23), 'learning_rate':'constant', 'learning_rate_init':0.7, 'max_iter':2000, 'random_state':42, 'solver':'sgd'}
	RandomForestClassifier	{'class_weight':'balanced', 'criterion':'gini', 'max_depth':5, 'n_estimators':51, 'random_state':42}
	svm.SVC	"{'C':1, 'class_weight':'balanced', 'gamma':'auto', 'kernel':'rbf', 'probability':True, 'random_state':42}
	DecisionTreeClassifier	"{'class_weight':'balanced', 'criterion':'gini', 'max_depth':4, 'random_state':42}
	GaussianNB	{ }
	KNeighborsClassifier	{'metric':'manhattan', 'n_neighbors':3, 'weights':'uniform'}
